# Astrocytes modulate sensory-evoked neuronal network activity

**DOI:** 10.1038/s41467-020-17536-3

**Published:** 2020-07-23

**Authors:** Justin Lines, Eduardo D. Martin, Paulo Kofuji, Juan Aguilar, Alfonso Araque

**Affiliations:** 1 Department of Neuroscience, University of Minnesota, 321 Church St SE, Minneapolis, MN 55455 USA; 2 Instituto Cajal, CSIC, Av. Dr. Arce, 37, 28002 Madrid, Spain; 3 Experimental Neurophysiology, Hospital Nacional de Parapléjicos SESCAM, Finca La Peraleda s/n, 45071 Toledo, Spain

**Keywords:** Neuroscience, Glial biology, Astrocyte, Sensory processing, Somatosensory system

## Abstract

While neurons principally mediate brain function, astrocytes are emerging as cells with important neuromodulatory actions in brain physiology. In addition to homeostatic roles, astrocytes respond to neurotransmitters with calcium transients stimulating the release of gliotransmitters that regulate synaptic and neuronal functions. We investigated astrocyte-neuronal network interactions in vivo by combining two-photon microscopy to monitor astrocyte calcium and electrocorticogram to record neuronal network activity in the somatosensory cortex during sensory stimulation. We found astrocytes respond to sensory stimuli in a stimulus-dependent manner. Sensory stimuli elicit a surge of neuronal network activity in the gamma range (30–50 Hz) followed by a delayed astrocyte activity that dampens the steady-state gamma activity. This sensory-evoked gamma activity increase is enhanced in transgenic mice with impaired astrocyte calcium signaling and is decreased by pharmacogenetic stimulation of astrocytes. Therefore, cortical astrocytes respond to sensory inputs and regulate sensory-evoked neuronal network activity maximizing its dynamic range.

## Abstract (web summary)

Astrocytes respond to neurotransmitters with calcium transients stimulating the release of gliotransmitters that regulate synaptic and neuronal functions. Here, the authors show that cortical astrocytes also respond to sensory inputs and regulate sensory-evoked neuronal network activity.

## Introduction

Cortical activity underlying sensory information processing is thought to be exclusively mediated by the electrical activity of neurons^1,2^. In the somatosensory cortex, inputs from peripheral stimulation elicit synchronized neuronal responses that contribute to generate particular oscillatory signatures in the electric field^3,4^. Neuronal network population activity results from intrinsic cellular properties, actions of neuromodulators and the level of synaptic inputs^5,6^. Among the different ranges of the cortical oscillatory spectrum, gamma oscillations (30–50 Hz) elicited by sensory inputs are thought to underly cortical information processing. Electrical signatures of neuronal network activity encompass the local field potential. However, electrically inexcitable cell types, like astrocytes, may influence neuronal network activity recorded in electrical fields.

Astrocytes, traditionally considered to play homeostatic roles without being directly involved in information processing, are emerging as non-neuronal cells that play important roles in brain physiology through bidirectional communication with neurons at tripartite synapses in different brain regions^7,8^. At the synapse level, astrocytes are known to respond with internal calcium elevations to a wide variety of synaptically released neurotransmitters^9^. In turn, astrocyte activation stimulates the release of neuroactive molecules, called gliotransmitters, that act on neuronal receptors to regulate neuronal activity and synaptic transmission and plasticity^10,11^. At network level, astrocytes in the hippocampus, cerebellum and neocortex have been shown to respond in vivo to sensory stimuli^12–14^ and to neuromodulators, such as acetylcholine^15,16^ or norepinephrine^17,18^. Moreover, astrocytes have been recently shown to influence hippocampal neuronal network activity^19^ and cortical up-states^20^. However, the properties of cortical astrocyte responses to sensory stimuli are poorly defined and their influence on sensory-evoked cortical neuronal network activity during sensory information processing remains unknown.

Therefore, while previous studies have shown the ability of cortical astrocytes to respond to sensory stimulation and contribute to cortical neuronal network states^12–15,21^, we here extend these observations by providing the quantification of sensory-evoked astrocyte responsiveness and the impact on sensory-evoked cortical neuronal network activity, quantitatively determining the parameters that define the relationship between sensory stimuli and cortical astrocyte-neuronal network responses.

We investigate these issues by combining in vivo two-photon astrocyte calcium imaging and electrophysiological recordings of neuronal network activity in the primary somatosensory cortex (S1), while delivering electrical stimuli to the hindpaw in anesthetized mice, which allows the selective stimulation of sensory inputs in a finely controlled manner. Here we show that the population of cortical astrocytes respond with high reproducibility to repeated sensory stimuli. Astrocytic somas and arborizations respond to the frequency, duration and intensities of the stimulation in a stimulus-dependent manner following sigmoidal curves. Furthermore, using simultaneous astrocyte calcium and electrocorticogram (ECoG) recordings, we show that sensory-evoked gamma power precedes correlated astrocyte network activity and produces a hysteresis pattern suggestive of a negative feedback loop. We also show that sensory stimuli evoke a transient rise in the gamma power that is followed by a decline to a steady-state phase that co-occurs with the delayed astrocyte calcium responses. Finally, the sensory-evoked gamma activity is decreased by specific pharmacogenetic activation of cortical astrocytes and is increased in a transgenic mouse with impaired astrocyte calcium activity. Therefore, cortical astrocytes respond to sensory inputs and control sensory-evoked cortical neuronal network activity, indicating that astrocytes are actively involved in cortical sensory information processing.

## Results

### Cortical astrocytes respond to sensory stimulation in vivo

**Fig. 1:**
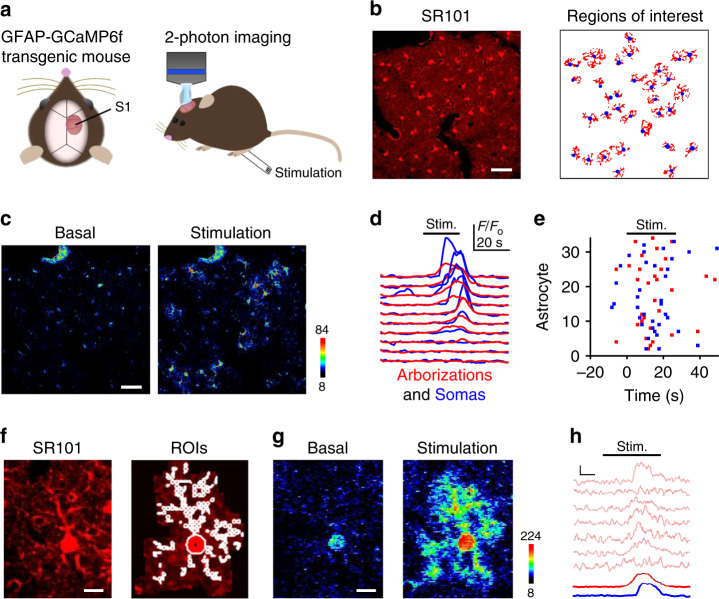
Two-photon astrocyte calcium imaging in vivo. **a** Craniotomy over the primary somatosensory cortex (S1) of GFAP-GCaMP6f transgenic mice to monitor stimulus-evoked astrocyte calcium using two-photon microscopy. **b** SR101-labeled astrocytes in vivo, and computationally determined regions of interest for somas (blue) and arborizations (red) using Calsee program (see Methods section). Scale bar = 50 µm. **c** Pseudocolor Ca^2+^ images of S1 astrocytes before and after hind paw stimulation. Scale bar = 50 µm. **d** Calcium traces of somas (blue) and arborizations (red). Horizontal black bar indicates sensory stimulation, as in other panels and figures. **e** Raster plot of somas (blue) and arborizations (red). **f** Astrocyte with regions of interest (ROIs). Scale bar = 10 µm. **g** Pseudocolor Ca^2+^ images before (basal) and during sensory stimulation. Scale bar = 10 µm. **h** Ca^2+^ traces from microdomains (pink), arborization (red), and the soma (blue). Scale = *F*/*F*_o_, 5 s. Example images and traces are representative of independent experiments carried out in 10 mice.

We have investigated the activity of astrocytes in layer 2/3 of the primary somatosensory cortex of adult transgenic GFAP-GCaMP6f mice in vivo (Fig. 1a) in response to sensory stimulation of the hindpaw. A craniotomy was centered over the cortical area representing the hindpaw (Fig. 1a) and astrocytes were identified with the fluorescent marker sulforhodamine SR101 (Fig. 1b) and their calcium activity was monitored using the genetically encoded calcium indicator GCaMP6f and two-photon microscopy imaging (Fig. 1c).

To test astrocyte responsiveness to sensory stimulation, we monitored astrocyte calcium dynamics before and after hindpaw stimulation with a 20 s duration train of electrical pulses (pulse duration: 0.5 ms; intensity: 2 mA; frequency: 2 Hz). Calcium elevations occurred in somas and cellular arborizations (i.e., astrocytic processes excluding the soma) of numerous astrocytes during peripheral stimulation, indicating that cortical astrocyte populations responded to somatosensory inputs (Fig. 1d–h). We next investigated whether the responsiveness of astrocyte cortical populations was reliably evoked by sensory stimuli. We monitored astrocyte activity while delivering five identical stimulation trains (intertrain interval: 2–3 min) (Supplementary Fig. 1a–c). About 50% of astrocyte arborizations (1-way ANOVA *p* < 0.001; Tukey HSD *p* < 0.01; *n* = 6 populations, 3 animals) and somas (1-way ANOVA *p* < 0.01; Tukey HSD *p* < 0.05; *n* = 6 populations, 3 animals) responded to all of the five stimuli, whereas a much lower proportion occasionally responded to some stimuli or did not respond at all (Supplementary Fig. 1d, e). These results indicate that cortical astrocytes responded to sensory stimulation with a high degree of network reliability.

### Sensory-evoked astrocyte Ca^2+^ dynamics is stimulus dependent

**Fig. 2:**
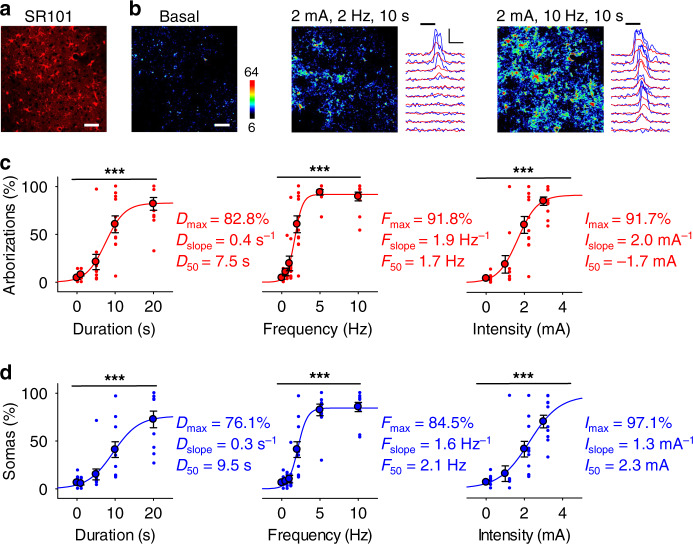
Astrocyte population responses are stimulus dependent. **a** SR101 staining. Scale bar = 50 µm. **b** Pseudocolor Ca^2+^ images with Ca^2+^ traces of arborizations (red) and somas (blue) before (basal) and during sensory stimulation with indicated parameters. Scale bar = 50 µm. Scale = Δ*F*, 10 s. Horizontal black bars indicate sensory stimuli. **c** Arborization responses vs. stimulus duration (*D*), frequency (*F*) and intensity (*I*), (1-way ANOVAs: duration: *p* = 4.7e^−13^; frequency: *p* = 5.3e^−23^; intensity: *p* = 2.0e^−10^; *n* = 11 imaging planes, 4 mice). Data were fit to the sigmoid function in Eq. (1) (see Methods section), where *D*_max_, *F*_max_, and *I*_max_ were the maximum responses for the respective stimulus parameter; *D*_slope_, *F*_slope_, and *I*_slope_ were the respective slopes, which are considered as indicative of the astrocyte discrimination of the sensory stimuli, i.e., how the astrocyte activity response changes as the stimulus changes; and *D*_50_, *F*_50_, and *I*_50_ were the stimulus parameter values at which astrocyte responses were 50% of the maximum values, which was considered as indicative of astrocyte sensitivity of sensory stimuli. **d** as in **c**, but for somas (1-way ANOVAs: duration: *p* = 1.0e^−10^; frequency: *p* = 3.3e^−21^; intensity: *p* = 1.6e^−7^; *n* = 11 imaging planes, 4 mice). Means ± SEM, ‘***’: *p* < 0.001, 1-way ANOVA. Representative images and traces are in line with independent experiments carried out in 4 mice. Source data are provided as a Source Data file.

We then characterized the dependence of the astrocyte network responses on the parameters of the sensory stimulus, by monitoring cortical astrocyte network activity (Fig. 2a, b) while delivering subsequent trains of stimuli (pulse duration: 0.5 ms; intertrain interval: 2–3 min) of varying durations (0–20 s), frequencies (0–10 Hz) and intensities (0–3 mA). Maintaining a constant intensity (2 mA) and frequency (2 Hz), the number of responding astrocytes, at both somas and arborizations, increased as the stimulus duration increased (1-way ANOVA: *p* < 0.001 for arborizations and *p* < 0.001 for somas; *n* = 11 populations, 4 animals; Fig. 2c, d). Values could be accurately fit to a sigmoid function (Eq. (1); see Methods section). For arborizations and somas, *D*_max_ was 82.8 and 76.1%, *D*_50_ was 7.5 and 9.5 s, and *D*_slope_ was 0.4 and 0.3 s^−1^, respectively. These results indicate that sensory-evoked astrocyte activity depends on the duration of the sensory stimulus.

Likewise, at a constant duration (10 s) and intensity (2 mA), the proportion of cortical astrocyte arborizations and somas increased as the stimulus frequency increased (1-way ANOVA: *p* < 0.001 for both arborizations and somas; *n* = 11 populations, 4 animals; Fig. 2c, d), following a sigmoidal function (Eq. (1); see Methods section), where *F*_max_ was 91.8 and 84.5%, *F*_50_ was 1.7 and 2.1 Hz, and *F*_slope_ was 1.9 and 1.6 Hz^−1^, for arborizations and somas, respectively. Similarly, at a constant duration (10 s) and frequency (2 Hz), the proportion of responding astrocyte somas and arborizations increased as the stimulation intensity increased (1-way ANOVA: *p* < 0.001 for arborizations and *p* < 0.001 for somas; *n* = 11 populations, 4 animals; Fig. 2c, d), according to the sigmoidal Eq. (1) (see Methods section), where *I*_max_ was 91.7 and 97.1%, *I*_50_ was 1.7 and 2.3 mA, and *I*_slope_ was 2.0 and 1.3 mA^−1^, for arborizations and somas, respectively. Taken together, these results indicate that the sensory-evoked cortical astrocyte network activation of somas and arborizations depends on the peripheral stimulation parameters, following sigmoidal curves.

### Astrocyte–neuron networks interact during sensory stimuli

**Fig. 3:**
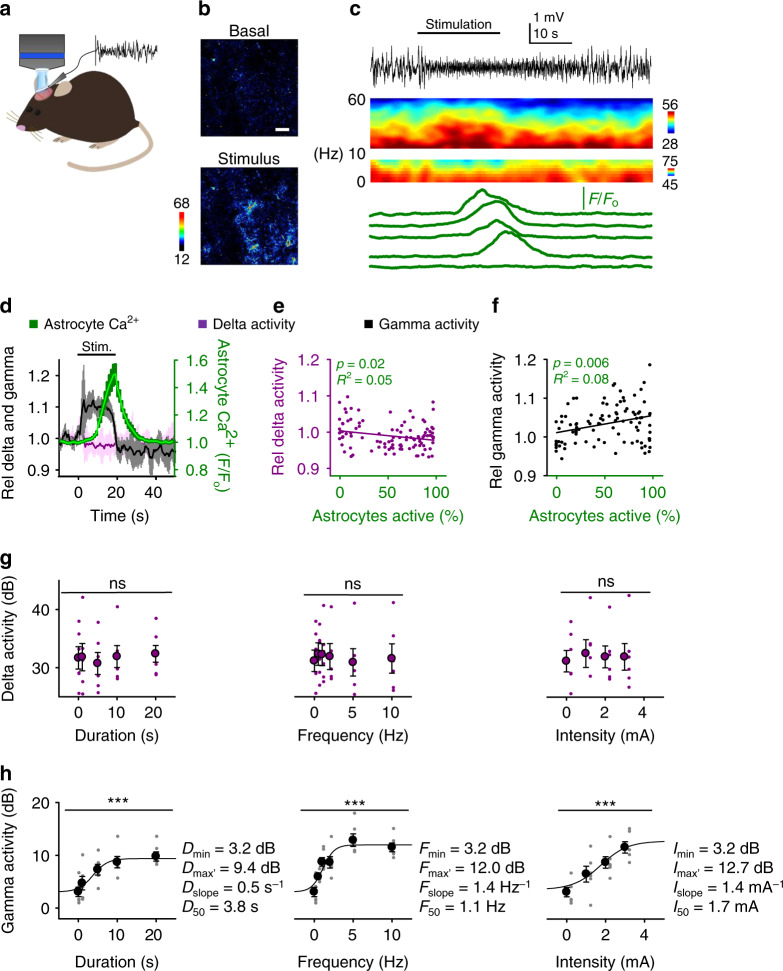
Cortical astrocyte activity co-occurs with increases in gamma activity during sensory stimulation. **a** Scheme of simultaneously recording astrocyte Ca^2+^ and ECoG in vivo. **b** Representative pseudocolor Ca^2+^ images of basal astrocyte activity and during hindpaw stimulation. Scale bar = 50 µm. **c** ECoG recording (black top trace), spectrograms (middle), and astrocyte Ca^2+^ levels (green bottom traces). **d** Astrocyte Ca^2+^ levels (green), relative gamma (30–50 Hz; black) and delta (0–4 Hz; purple) activity vs. time. Lines and shadows represent mean and SEM, respectively, as in other figures. **e** Relative delta activity vs. percentage of active astrocytes (*n* = 100 imaging planes, 3 animals). **f** as **e** but for relative gamma activity instead of relative delta activity. **g** Steady-state delta activity vs. the duration (*D*; left), frequency (*F*; center) and intensity (*I*; right) of the sensory stimulation (1-way ANOVA: duration: *p* = 0.21, frequency: *p* = 0.49, intensity: *p* = 0.17; *n* = 6 animals). **h** as **g**, but gamma activity instead of delta activity (1-way ANOVA: duration: *p* = 0.0005, frequency: *p* = 1.0e^−7^, intensity: *p* = 0.0004; *n* = 6 animals). Data were fit to the sigmoid function in Eq. (2) (see Methods section), where *D*_max_, *F*_max_, and *I*_max_ were the response maximum values over *D*_min_, *F*_min_, and *I*_min_ (i.e., the minimum value of the respective stimulus parameter), *D*_slope_, *F*_slope_, and *I*_slope_ were the respective slopes; and *D*_50_, *F*_50_, and *I*_50_ were the stimulus parameter values at which responses were 50% of the maximum values. Mean ± SEM. ns: *p* > 0.05 and ***: *p* < 0.001 using 1-way ANOVA. Source data are provided as a Source Data file.

Previous results show that astrocyte network activity is regulated by sensory stimuli, which are known to regulate spontaneous neuronal cortical activity^20,22^. We then investigated whether astrocyte and neuronal network activities were associated. We recorded the neuronal population local field potential via electrocorticogram (ECoG) and simultaneously monitored astrocyte calcium activity (Fig. 3a–c), before and during hindpaw stimulation. Sensory stimulation elicited a rapid change in cortical neuronal network activity, manifested in the ECoG and the spectrogram of the frequency bands, and a delayed elevation in the astrocyte calcium population activity (Fig. 3c). We then analyzed the relationship between the astrocyte calcium activity and the different frequency bands of the ECoG.

Sensory stimulation elicited a rapid and transient increase of the cortical delta (0–4 Hz) activity that shortly receded to a steady-state below baseline prior to astrocyte calcium activation (Fig. 3d). Across several stimuli and preparations, a negative correlation was found between sensory-evoked cortical delta activity changes and the proportion of active astrocytes (Student’s *t*-test of a Pearson’s correlation: *p* < 0.05, *R*^2^ = 0.05; *n* = 100 imaging planes, 3 animals; Fig. 3e). In addition to this relation between delta activity and astrocyte calcium activities, different stimulus durations, frequencies, and intensities, which elicited stimulus-dependent astrocyte responses (see Fig. 2), did not significantly alter the steady-state delta activity (1-way ANOVA: duration: *p* = 0.21, frequency: *p* = 0.49, intensity: *p* = 0.17; *n* = 6 animals; Fig. 3g). Other relatively low-frequency bands of the ECoG, i.e., slow-wave (0–1 Hz) and theta (4–8 Hz) activities, were not correlated with the astrocyte population activity (Supplementary Fig. 2a–f). These results indicate that sensory-evoked low-frequency and theta were not associated with astrocytes, and delta was negatively correlated.

The cortical gamma (30–50 Hz) activity rapidly increased after initiation of the sensory stimulation, persisted throughout the stimulus duration, and co-occurred with the elevation of astrocyte calcium (Fig. 3d). We found a significant correlation between the relative increases in gamma activity and the proportion of responding astrocytes (Student’s *t*-test of a Pearson’s correlation *p* < 0.01; *R*^2^ = 0.08; *n* = 100 imaging planes, 3 animals; Fig. 3f). In addition, relative cortical gamma power changed by varying the stimulus duration, frequency and intensity (1-way ANOVA: duration: *p* < 0.001, frequency: *p* < 0.001, intensity: *p* < 0.001; *n* = 6 animals; Fig. 3h); similar to the observed stimulus-dependent astrocyte population responses (see Fig. 2). We fit these data to a sigmoid function in Eq. (2). Likewise, beta (13–30 Hz) activity, which displayed a persistent sensory-evoked increase and stimulus-dependence, also positively correlated with the astrocyte activity (Supplementary Fig. 2j–l). Altogether, these results suggest that, in contrast with low frequencies, the enhancement of high frequency network activity evoked by peripheral stimulation was positively correlated with the astrocyte population activity elicited by the sensory stimuli. During the 20 s stimulation, the gamma (and beta) activities declined at the end of the stimulus, coinciding with the maximum astrocyte calcium response (Fig. 3d and Supplementary Fig. 2j).

**Fig. 4:**
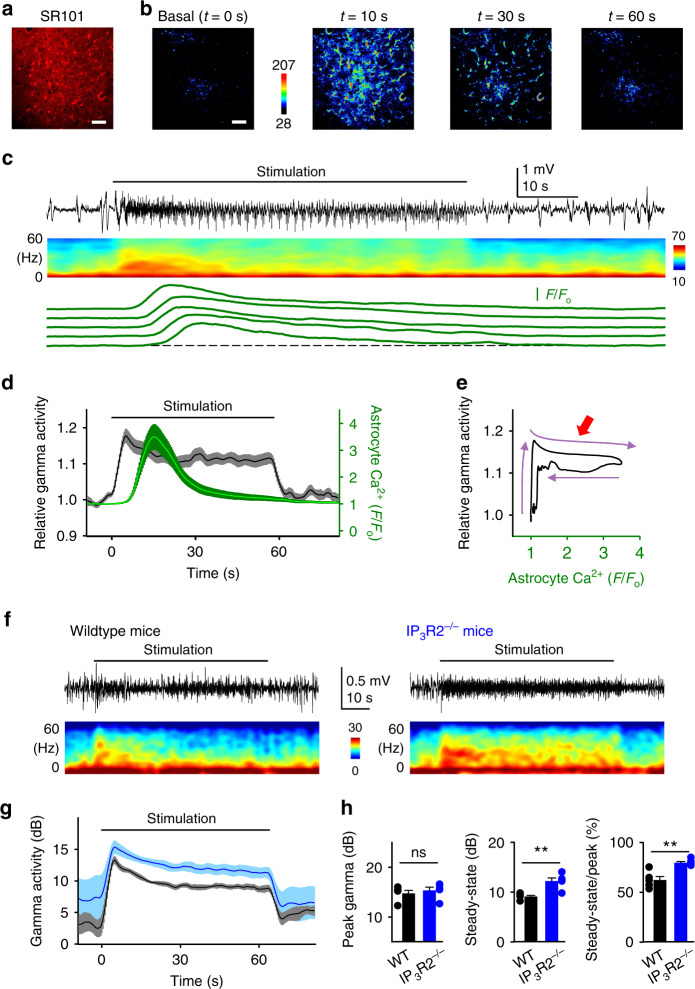
Astrocyte–neuron interactions revealed during sustained sensory stimulation. **a** SR101 staining. Scale bar = 50 µm. **b** Representative pseudocolor Ca^2+^ images of basal astrocyte activity and during hind paw stimulation at indicated times from the stimulus onset. Scale bar = 50 µm. **c** ECoG recording (black top trace), spectrogram (middle), and astrocyte Ca^2+^ levels (green bottom traces). **d** Relative gamma activity (black) and relative astrocyte Ca^2+^ levels (green) over time; horizontal black bar indicates sensory stimulation (2 mA, 5 Hz, 60 s). Lines and shadows represent mean and SEM, respectively, as in other panels. **e** Relative gamma activity vs. astrocyte Ca^2+^ levels. Note the hysteresis loop between neuronal network activity and astrocyte calcium denoted by purple arrows, and the gamma activity decrease following the increase in astrocyte Ca^2+^ levels (red arrow). **f** Extracellular recordings (black top traces) and spectrograms (bottom) from wildtype (left) and *IP*_3_*R2*^−/−^ (right) mice. **g** Gamma activity vs. time from wildtype (black) and *IP*_3_*R2*^−/−^ (blue) mice in response to sustained sensory stimulation. **h** Peak gamma response to stimulation (left; *p* = 0.59; *n* = 5 animals each), steady-state gamma activity after 50 s of the stimulation (center; *p* = 0.003) and the ratio of steady-state over peak gamma (right; *p* = 0.002). Mean ± SEM. ns: *p* > 0.05 and **: *p* < 0.01 using two-sided student *t*-test. Images and traces are representative of data obtained independently across 5 mice. Source data are provided as a Source Data file.

To test whether astrocyte calcium may contribute to this decline, we stimulated the hindpaw with a longer-lasting stimulus (60 s; Fig. 4a–c). Consistent with above described results, sensory stimulation elicited both the rapid enhancement of gamma activity and the delayed astrocyte activation (Fig. 4c). Notably, paralleling the astrocyte population calcium rise, the gamma activity partially diminished to a steady-state that lasted until the stimulus ended (Fig. 4d). The plot of the sensory-evoked astrocyte calcium versus cortical gamma activity displayed a marked hysteresis, i.e., a change in evoked gamma activity as the astrocyte calcium changes (Fig. 4e), suggesting that astrocyte activity might contribute to regulate sensory-evoked gamma network activity.

To test this idea, we analyzed the changes in stimulus-evoked gamma activity in *IP*_*3*_*R2*^−/−^ mice, in which calcium activity is largely impaired in astrocytes^23^. We first confirmed in parallel experiments that *IP*_*3*_*R2*^−/−^ mice displayed strongly diminished astrocyte activity at the microdomain level both in basal and during sensory stimulation (wildtype mice: 1.6 ± 0.6 and 3.1 ± 0.4 events/min in basal and during stimulation, respectively; *p* < 0.01; *IP*_*3*_*R2*^−/−^ mice: 0.8 ± 0.2 and 1.1 ± 0.2 events/min in basal and during stimulation, respectively; *p* < 0.01; *n* = 5 imaging planes, 2 animals; Supplementary Fig. 3n). Moreover, astrocyte calcium activity was negligible at the network scale of arborizations and somas (wildtype mice arborizations: 1.0 ± 0.3 and 3.1 ± 0.2 events/min in basal and during stimulation, respectively; *p* < 0.001; *IP*_*3*_*R2*^−/−^ mice arborizations: 0.2 ± 0.1 and 0.5 ± 0.2 in basal and during stimulation, respectively; *p* = 0.15; *n* = 5 imaging planes, 2 animals; wildtype mice somas: 1.1 ± 0.3 and 2.7 ± 0.2 events/min in basal and during stimulation, respectively; *p* < 0.05; *IP*_*3*_*R2*^−/−^ mice somas: 0.4 ± 0.1 and 0.5 ± 0.1 in basal and during stimulation, respectively; *p* = 0.18; *n* = 5 imaging planes, 2 animals; Supplementary Fig. 3o–p). These results, which are in agreement with previous reports (cf.^15,24–26^), support the validity of *IP*_*3*_*R2*^−/−^ mice as a loss-of-function approach.

*IP*_*3*_*R2*^−/−^ mice showed heightened basal gamma activity compared to wildtype (3.5 ± 1.0 dB in wildtype vs. 9.0 ± 1.8 dB in *IP*_*3*_*R2*^−/−^ mice; *p* < 0.05; *n* = 4 animals; Supplementary Fig. 4), and, like wildtype mice, responded to sustained sensory stimulation with the initial surge of gamma activity that declined to a persistent steady state (Fig. 4f–g). However, while the gamma showed similar peak values in wildtype and *IP*_*3*_*R2*^−/−^ mice (14.7 ± 0.7 dB and 16.2 ± 1.1 dB, respectively; *p* = 0.59; *n* = 5 animals each; Fig. 4h), it reached higher steady-state values in *IP*_*3*_*R2*^−/−^ mice (steady-state: 9.1 ± 0.3 dB in wildtype vs. 12.2 ± 0.7 dB in *IP*_*3*_*R2*^−/−^; *p* < 0.01; Fig. 4h). Moreover, the temporal decline of gamma activity during the sensory stimulation was significantly diminished in *IP*_*3*_*R2*^−/−^ mice, as evidenced by the steady-state/peak ratios (62.2 ± 3.7% in wildtype vs. 79.5 ± 1.4% in *IP*_*3*_*R2*^−/−^ mice; *p* < 0.01; Fig. 4h). Taken together, these results show that the dynamic range of the gamma activity is reduced in *IP*_*3*_*R2*^−/−^ mice, and indicate that astrocyte activity regulates the steady-state of the sensory-evoked gamma network activity.

### Astrocytes regulate sensory-evoked neuronal network activity

**Fig. 5:**
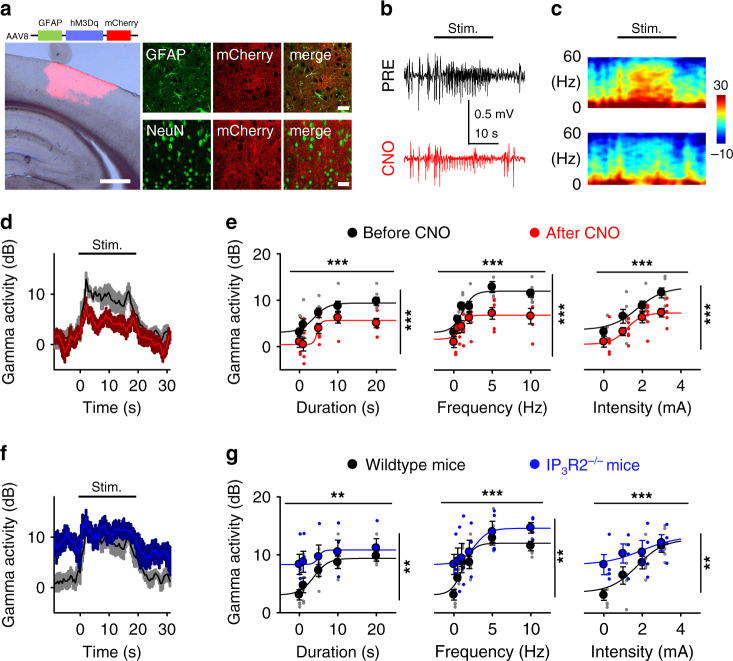
Astrocytes modulate neuronal network response to sensory stimulation. **a** Viral expression of DREADDs in S1 astrocytes. Scale bars = 500 µm, 10 µm. **b** ECoG recordings during hind paw stimulation before (black top trace, PRE) and after (red bottom trace, CNO) i.p. injection of CNO. Images and traces are representative of independent experiments from 6 mice. **c** Spectrograms of **b**. **d** Gamma activity vs. time before (black) and after CNO (red). Lines and shadows represent mean and SEM, respectively, as in other panels. **e** Gamma activity vs. the duration, frequency and intensity of the sensory stimulation before (black) and after CNO (red) injection (duration: 2-way ANOVA for stimulus: *p* = 1.9e^−6^, for CNO: *p* = 1.3e^−5^; frequency: 2-way ANOVA for stimulus: *p* = 1.9e^−8^, for CNO: *p* = 1.1e^−6^; intensity: 2-way ANOVA for stimulus: *p* = 2.9e^−7^, for CNO: *p* = 3.4e^−4^; *n* = 6 animals). Data were fit to the sigmoid function in Eq. (2) (see Methods section; see Supplementary Table 2 for fitting parameters). **f**–**g**, as **d**–**e**, but in wildtype (black) and *IP*_*3*_*R2*^−/−^ (blue) mice (duration: 2-way ANOVA for stimulus: *p* = 0.001, for genotype: *p* = 0.001; frequency: 2-way ANOVA for stimulus: *p* = 1.3e^−7^, for genotype: *p* = 0.002; intensity: 2-way ANOVA for stimulus: *p* = 0.0004, for genotype: *p* = 0.007; *n* = 6 animals). Note that data of before CNO and wildtype are the same. Mean ± SEM. ‘***’: *p* < 0.001 and ‘**’: *p* < 0.01 for 2-way ANOVA. Source data are provided as a Source Data file.

Above results suggest that sensory-evoked astrocyte and neuronal network activities are associated, but do not provide cause-effect evidence. To evaluate whether astrocytes can regulate cortical neuronal network responses to sensory stimuli, we specifically activated cortical astrocytes using pharmacogenetics while monitoring the local field potential during sensory stimulation. Virus (AAV8-GFAP-hM3Dq-mCherry), which contained the hM3Dq (Gq) Designer Receptors Exclusively Activated by Designer Drugs (DREADDs) under the astrocyte *GFAP* promoter and the mCherry tag to localize viral infection, were stereotaxic injected into the primary somatosensory cortex. The selective DREADD expression by astrocytes in the primary somatosensory cortex was confirmed by immunohistochemistry (Fig. 5a). Immunohistochemical analysis indicated that DREADD expression (monitored by the expression of the fluorescent reporter mCherry) vastly occurred in astrocytes; indeed, while 95.4% of astrocytes were positive for GFAP and mCherry (*n* = 130 cells, 9 slices) only 6.2% of neurons were positive for NeuN and mCherry (*n* = 259 cells, 4 slices). Intraperitoneal injection of the DREADD selective agonist Clozopine N-Oxide (CNO)^27^ led to an increase in astrocyte calcium (from 12.1 ± 1.5% to 45.3 ± 8.5% active astrocytes; *p* < 0.05; *n* = 4 animals; Supplementary Fig. 5) compared to control conditions with a virus lacking DREADDs (i.e., AAV8-GFAP-mCherry) (from 13.8 ± 6.5% to 11.6 ± 4.2% active astrocytes; *p* = 0.58; *n* = 4 animals) or saline injections (from 16.6 ± 2.3% to 22.3 ± 6.6% active astrocytes; *p* = 0.74; *n* = 3 animals).

While recording the local field potential, we stimulated the hindpaw before and 30 min after intraperitoneal injection of CNO (Fig. 5b, c). Consistent with above results, before CNO injection, sensory stimulation of virus-infected mice increased the gamma cortical activity in a stimulus parameter-dependent manner (Fig. 5d, e), increasing as the stimulus duration, frequency or intensity increases. Activation of DREADD-expressing astrocytes with CNO dampened cortical gamma responses to hindpaw stimulation (Fig. 5d, e). Notably, the stimulus-dependence of cortical gamma activity remained, varying as the stimulus duration, frequency, and intensity changed. However, gamma response profiles were significantly reduced across all the stimulating parameters following CNO injections (duration: 2-way ANOVA for stimulus: *p* < 0.001, for CNO: *p* < 0.001; frequency: 2-way ANOVA for stimulus: *p* < 0.001, for CNO: *p* < 0.001; intensity: 2-way ANOVA for stimulus: *p* < 0.001, for CNO: *p* < 0.001; *n* = 6 animals; Fig. 5e). Indeed, the stimulus-dependent gamma activity curves were shifted to lower values upon CNO application, as indicated by the lower *D*_max_, *F*_max_, *I*_max_, *D*_min_, *F*_min_, and *I*_min_ in CNO when compared to control values (Supplementary Table 2), indicative of a general reduction of the sensory-evoked gamma activity under astrocyte activation. These alterations occur without changes in the astrocyte discrimination or sensitivity to the stimuli, as indicated by the similar values of *D*_slope_, *F*_slope_, and *I*_slope_, and *D*_50_, *F*_50_, and *I*_50_, respectively (Supplementary Table 2).

As controls, and to account for potential CNO off-target effects^28^, we injected CNO into animals that received stereotaxic surgery of the virus AAV8-GFAP-mCherry, i.e., with no DREADDs. We did not observe a significant difference in any of our stimulation responses before or after injections of CNO in animals that received control viral injections (2-way ANOVAs for CNO: duration: *p* = 0.43; frequency: *p* = 0.10; intensity: *p* = 0.24; *n* = 3 animals; Supplementary Fig. 6a). In addition, we found that astrocyte-specific DREADDs expression also did not account for the observed change in gamma response to sensory stimulation without the inclusion of CNO, when we compared responses before and after saline injections (2-way ANOVAs for saline: duration: *p* = 0.27; frequency: *p* = 0.21; intensity: *p* = 0.07; *n* = 3 animals; Supplementary Fig. 6b). This further confirms that the observed reductions in sensory-evoked gamma activity was due to CNO specifically activating DREADDs selectively expressed in astrocytes in S1.

Finally, we evaluated the electrophysiological response profile of *IP*_*3*_*R2*^−/−^ mice that have largely abated calcium activity due to the genetic knock out of astrocytic type-2 IP_3_ receptors (Supplementary Fig. 3), which are key elements in the G protein coupled-receptor-mediated calcium mobilization in astrocytes. Across a battery of stimulations, we found that cortical gamma was increased in *IP*_*3*_*R2*^−/−^ mice compared to wild type controls (duration: 2-way ANOVA for stimulus: *p* < 0.01, genotype: *p* < 0.01; frequency: 2-way ANOVA for stimulus: *p* < 0.001, genotype: *p* < 0.01; intensity: 2-way ANOVA for stimulus: *p* < 0.001, genotype: *p* < 0.01; Fig. 5f–g; see Supplementary Table 2 for fitting parameters to Eq. (2) defining the stimulus-dependency), suggesting that astrocyte calcium activity contributes to the regulation of sensory-evoked cortical network activity.

Taken together these data suggest that astrocytes regulate cortical network activity, restraining the sensory-evoked increases of cortical gamma.

## Discussion

Through the combined use of two-photon microscopy and ECoG recordings in vivo we identified temporal and mechanistic links between sensory-evoked responses in astrocyte and neuronal activity, indicating astrocyte—neuronal network responsiveness to sensory stimulation. While previous studies have reported on cortical astrocyte responses to somatosensory stimulation and astrocyte influence on cortical neuronal network states^12–15,21^, present results provide the first quantification of the sensory-evoked astrocyte responsiveness and the impact on sensory-evoked cortical neuronal network activity. We found cortical astrocytes exhibit stimulus-dependent and reliable responses to sensory stimulation. Calcium rises in astrocyte networks were found to be temporally associated with changes in neuronal network activity assessed by the spectral content of ECoG. Finally, we were able to identify downstream astrocyte neuromodulation of sensory-evoked neuronal network responsiveness by incorporating astrocyte-specific DREADDs activation of astrocytes and conversely transgenic mice with impaired astrocyte calcium signaling. Therefore, our results indicate that astrocytes are involved in the modulation of neuronal population excitability in the somatosensory cortex during responses to sustained peripheral inputs (or peripheral stimulation).

While qualitative astrocyte responsiveness to sensory stimuli has been shown in the visual and barrel cortex and the olfactory bulb in vivo^12,29,30^, our results provide a quantification of the astrocyte responses, establishing stimulus-response curves with three defined parameters. The dependency of astrocyte responsiveness to the stimulus frequency agrees with the fact that changes in the frequency of peripheral nerve stimulation lead to increased firing of single unit neuronal activity in the cortex^31^, indicating that cortical astrocytes follow the sensory information encoded by neuronal inputs to the cortex. On the other hand, because different intensities of sensory stimulation are known to activate different amounts of peripheral fibers^32^, the intensity-dependent astrocyte responsiveness indicates that the spatial extension of the activated astrocytic population depends on the volume of neuronal inputs activated. Finally, the stimulus-duration dependency indicates that astrocyte responses follow the duration of the sensory input to the cortex. The stimulus-dependent response is an important property of information processing by cortical neuronal networks^2^. Present quantitative results show that stimulus-dependence is displayed by the responsiveness in the population of cortical astrocytes as well.

A recent study in awake animals has shown that locomotion triggers simultaneous activation of astrocytes in multiple brain regions, including the visual cortex^18^, while anesthesia is known to decrease astrocyte activity^33^. The use of anesthetized animals in this study prevented confounding effects produced during locomotion and allowed the controlled sensory stimulation of somatosensory inputs. Furthermore, urethane is widely used to study cortical neuronal population activity^20,22,34^. Nevertheless, even if the absolute astrocyte excitability is reduced under our experimental conditions, the relative stimulus-response curves are not expected to be altered. On the other hand, the electrical stimulation used in this study, which allowed a fine experimental control, may be limited as a natural somatosensory stimulation. However, several reports indicate that this type of stimulation produces a reliable recruitment of the amount and modalities of somatosensory peripheral inputs^32,35^.

Cortical states are related to different levels of neuronal activity during natural behavioral states, which are identified by oscillatory features (ranges of frequencies and amplitudes) in the electrical field from neuronal populations at the cortical level monitored by ECoG^4,6^. Like astrocyte responses, neuronal network gamma activity too displayed stimulus-dependent activation. In agreement with previous reports^36,37^, we observed that sensory stimulation elicited alterations in the gamma range (30–50 Hz). Stimulus-response curves of sensory-evoked gamma activity matched those of astrocyte calcium responses. Furthermore, our data shows the existence of negative and positive correlation between astrocyte activity and delta and gamma activities, respectively (Fig. 3e, f). The statistically significant correlation indicates that these network states are associated with the astrocyte calcium activity, but the relatively low values of the correlation coefficients suggest the involvement of additional factors. Altogether, these findings suggest that cortical astrocyte and neuron populations both respond to sensory stimulation in a coordinated manner.

After determining the stimulus-dependency of the astrocyte and neuronal network responses (Figs. 1–3), a strong long-lasting stimulus was designed to test the regulatory feedback signaling provided by astrocytes (Fig. 4). The results obtained by delivering a more prolonged stimulus also indicate that astrocytes contribute to the cortical sensory adaptation. Indeed, the decline of cortical gamma activity associated with the delayed astrocyte calcium rise and the hysteresis relationship between gamma activity and astrocyte calcium suggests the astrocyte regulation of sustained gamma activity. The responses to this type of stimulation is likely to involve additional time-dependent desensitization and adaptation processes. While further studies are required to determine the involvement of additional neuronal mechanisms, our results support the idea that astrocyte activity regulates the steady-state of the sensory-evoked gamma network activity. This idea is further supported by the decreased steady-state of gamma activity elicited by the prolonged stimulus in wildtype compared to *IP*_*3*_*R2*^−/−^ mice with impaired astrocyte calcium signaling. The fact that the decline in gamma activity was not abolished in *IP*_*3*_*R2*^−/−^ mice is not surprising, considering that intrinsic neuronal and synaptic mechanisms are also present^38^.

Cortical gamma activity is believed to play a major role in sensory integration and attention^39,40^. Gamma has been posited to underly sensory binding, but critics of this theory denote gamma activity as merely a signature of neuronal activity^41^. Regardless of the theoretical framework, gamma is generally considered to represent the large-scale appearance of neuronal network activity, in which neurons are in a more excitable state in opposition to lower ranges of frequencies in which neurons are less excitable. Our work adds to this debate by presenting a sensory-evoked dose dependence of cortical gamma activity that was absent in a different range of cortical frequencies, namely delta, correlated to a lower level of neuronal excitability. And we go a step further by including that astrocytes are also involved in this network synchrony and indeed play an important regulatory role. Transgenic *IP*_*3*_*R2*^−/−^ mice, missing a key element in astrocyte calcium signaling, were shown to have increased levels of sensory-evoked gamma activity, as well as baseline ECoG activity. In contrast, specific pharmacogenetic activation of astrocytes, which was found to increase their calcium, was found to dampen the sensory-stimulation evoked gamma-power. Both experimental protocols resulted in a smaller range of gamma activity; whether constrained to lower levels by DREADD activation, or unleashed to heightened levels in animals with impaired astrocyte activity. Taken together, this suggests cortical astrocytes actively regulate the dynamic range of cortical neuronal network excitability that is responsible for sensory-evoked cortical gamma activity. Therefore, astrocytes are involved in the regulation of the level of excitability for the somatosensory system during information processing.

Present results show interesting aspects of the temporal dynamics of the sensory-evoked astrocyte responses and gamma activity regulation. First, astrocyte Ca^2+^ elevations display a several seconds delay from the onset of the stimulus. The reason for this is unknown, but it is not surprising considering that synaptically-evoked astrocyte Ca^2+^ elevations are largely mediated by the activation of G protein-coupled receptors and *IP*_*3*_*R*_*2*_-dependent Ca^2+^ mobilization from internal stores^9,42^, which presents relative slower time courses than neuronal activation through ligand-gated and voltage-gated channels. Second, during the long-lasting stimulation (Fig. 4d), the Ca^2+^ signal subsided while the gamma activity displayed a steady-state. While astrocyte Ca^2+^ did reduce, it remained above baseline until the end of the stimulation similar to gamma levels. Perhaps the downstream signaling processes triggered by the astrocyte Ca^2+^ was persistent or had persistent effects on the neuronal network. Nevertheless, further studies are required to identify the mechanisms that account for these temporal observations.

Multiple signaling pathways may be putatively involved in the bidirectional astrocyte-neuron communication underlying the observed results. On the one hand, astrocytes are known to respond with Ca^2+^ elevations to a wide variety of neurotransmitters released at cortical synapses active during the sensory stimulation, including glutamate, GABA, acetylcholine, norepinephrine or dopamine^9,11,42,43^. On the other hand, multiple astrocyte signaling mechanisms may influence neuronal activity^9,11,44^, either through synapse-specific or volume transmission^45–47^. Astrocytes activated during sensory stimulation may release transmitters, such as glutamate^48,49^, GABA^50^, or ATP/adenosine^22,51^, that may affect neuronal activity, as well as excitatory and inhibitory synapses. In addition, astrocytes can modulate neuronal activity through chemical homeostasis mechanisms involving inward K^+^ currents or neurotransmitter clearance at the synaptic cleft^29,52^. Finally, astrocytes could alter neuronal activity using their more traditional support mechanisms such as metabolic clearance and neurovascular coupling^29,53^. While further studies out of the scope of the present work are needed to identify the exact regulatory mechanisms involved, our data suggests a dynamical link between astroglial-neuronal networks during sensory information processing in the cortex.

Proper cortical harmony is necessary for healthy brain function. Changes in frequency band power have become a metric for sensory perception. However, alterations in these spectral changes present in patients suffering from neurological disorders^54^. Most notable is the unwarranted seizures of electrical activity in epilepsy^55^. In contrast, Schizophrenia patients have been shown to have lowered sensory-evoked gamma activity, suggesting alternatively improper network synchrony^56^. In addition, neurodegenerative diseases such as Alzheimer’s disease and Parkinson’s disease have altered gamma activity^57,58^. Astrocytes have long been held to play solely homeostatic mechanisms, many of which are challenged in neurological disorders^59^. Our findings suggest astrocytes regulate operational neuronal network activity. If astrocytes are active participants in both environmental and activity homeostasis, then it would be natural to suppose astrocytes may be targets for future diagnostic and therapeutic strategies in the care of neurological disorders^7^. The provided quantification of the stimulus-responses in astrocytes may be useful for future studies investigating alterations of sensory information in animal models of brain disorders, like schizophrenia or Alzheimer´s disease.

In conclusion, present results show that astrocytes in the primary somatosensory cortex reliably respond with calcium elevations to sensory stimuli in a stimulus-dependent manner, following stimulus-response curves with quantified defining parameters. Sensory-evoked astrocyte activity strongly correlated with gamma neuronal network activity, but not or minimally with other frequency bands (low-frequency, delta, theta, alpha and beta). Furthermore, astrocyte activity controlled the magnitude and dynamic range of sensory-evoked gamma activity underlying sensory information processing. Therefore, we conclude that sensory information processing in the primary somatosensory cortex involves the coordinated activity of cortical astrocytes and neurons.

## Methods

### Animal use and care

All the procedures for handling and sacrificing animals were approved by the University of Minnesota Institutional Animal Care and Use Committee (IACUC) in compliance with the National Institutes of Health guidelines for the care and use of laboratory animals. We used both female and male animals that were 2–6 months of age, kept on a continuous 12 h light/dark cycle, at temperatures between 68–74° at 30–70% humidity, and freely available to food and water.

### Stereotaxic surgery

Mice were anesthetized with 1.8 mg/kg urethane administered intraperitoneally (IP). Anesthetized mice were placed in a stereotaxic atop a heating pad controlled with an anal probe feedback to maintain body temperature, and faux tears were applied to prevent corneal dehydration. An incision was made down the midline of the scalp and the skin was parted to expose the skull. Screws were placed over the right frontal plate and interparietal plate. In experiments recording electrocorticogram (ECoG), the interparietal screw was soldered to a reference wire. A craniotomy was made no more than 2 mm in diameter centered over the primary somatosensory cortex (S1; in mm from bregma: −1_a–p_, 1.5_m–l_)^60^. After the dura was removed, sulforhodamine 101 (SR101) was topically applied to the exposed cortex to label astrocyte structure (50 µM for 20 min)^61^, i.e., an experimental protocol that does not affect cortical activity^61^. Agarose (1%) was made from artificial cerebrospinal fluid (containing in mM: NaCl 140, KCl 5, MgCl_2_ 1, CaCl_2_ 2, EDTA 1, HEPES-K 8.6, Glucose 10) and placed on the exposed cortex before fixing a glass coverslip over the craniotomy using dental cement. To record ECoG, a 0.25 mm tungsten wire was placed over the exposed cortex. Finally, a frame was mounted onto the exposed skull using dental cement.

### In vivo two-photon calcium fluorescence imaging

GFAP-GCaMP6f mice, obtained by crossing transgenic mice expressing the *Cre* recombinase under the control of the astrocyte promoter *GFAP* (GFAP-Cre mice) with mice carrying the floxed-STOP cassette upstream of the widely expressed GCaMP6f; and *IP*_*3*_*R2*^−/−^ mice, injected into S1 with AAV5-GfaABC1d-GCaMP6f, were used. In vivo imaging was performed by placing the mouse underneath a Leica SP5 multiphoton upright microscope. Videos were obtained for 60 s at either 256 × 256 or 512 × 512 resolution with a sampling interval of 0.2–0.5 s. Red and green fluorescence was obtained in parallel to match red SR101-stained astrocyte structure with green GCaMP6f astrocyte calcium. In addition to astrocytes, SR101 has been shown to label oligodendrocytes as well^62–64^. However, while we used SR101 labeling to structurally define cells, the cellular Ca^2+^ activity was assessed using GCaMP6f, which was selectively expressed under the astroglial promoter *GFAP*, supporting the idea that the recorded calcium activity derives from astrocytes. Hindpaw stimulation was delivered across two needles placed in the hindpaw of the animal.

### In vivo electrophysiology

Cortical local field potential (ECoG) recordings were done using a tungsten micro-electrode placed 100–300 µm below the cortical surface into layer 2/3 of the somatosensory cortex. Reference and ground were recorded from a wire soldered to a screw in the interparietal plate. Electrophysiological recordings were sampled at 10 kHz using an A-M Systems model 3000 ACDC differential amplifier, band pass filtered between 1–3000 Hz, and digitized using an Axon Digidata 1550 A acquisition system connected to a PC and monitored with AxoScope software (pCLAMP; AxoScope version 10.5.0.9). Before delivering sensory stimulation protocols, electrophysiological recordings were monitored for up to 30 min for stability, i.e., maintained activity in the low frequency range with occasional regular transient activity in the gamma range (Supplementary Fig. 7). The regular transient events were identified as up-states according to their amplitude (0.17 ± 0.05 mV) duration (0.93 ± 0.10 s) and waveform pattern (Supplementary Fig. 7c). The few animals that did not show stable recordings were discarded. To ensure cortical activity was restored to baseline levels following stimulation, 2–3 min of interstimulus times were taken before the subsequent stimulation was performed.

### Electrical stimulation

A bipolar electrode needle was placed on both sides of the wrist of the hindpaw contralateral to the recorded cortical hemisphere. Square electrical pulses with 0.5 ms duration and increasing intensities (1, 2, 3 mA) and variable frequencies (0.5, 1, 2, 5, 10 Hz) were applied in sustained durations (1, 5, 10, 20, 60 s). Stimulus parameters were combined in various ways to differentially activate neuronal populations in the neocortex with the purpose to characterize different levels of activation of astrocytes. In this sense, increasing intensity allowed the recruitment of different amounts of peripheral neuronal fibers, therefore increasing intensities were used to increase the cortical neuronal population activity. Increasing frequencies were used to produce increasing synaptic activity in the neuronal cortical population to test astrocyte activation depending on synaptic frequencies. Different durations of sustained stimulation were used in order to overpass the required time for astrocyte activation/responses.

### AAV viral surgeries

Animals were anesthetized using a ketamine (10 mg/mL) xylazine (1 mg/mL) mixture and placed on a heating pad to maintain body temperature and faux tears were applied to the cornea. Animals were placed in a stereotaxic apparatus and an incision was made down the midline of the scalp to expose the skull. A hole was drilled over the somatosensory cortex (S1: −1_a–p_, 1.5_m–l_), and a Hamilton syringe was lowered to (in mm from bregma: −0.7 and −0.9_d–v_) and 1 µL of AAV8-GFAP-hM3Dq-mCherry virus was injected bilaterally at 100 nL/min. Mice were then sutured and left to heal for 2–3 weeks. In control conditions, a virus of AAV8-GFAP-mCherry was injected instead. To image astrocyte Ca^2+^ in *IP*_*3*_*R2*^−/−^ mice or wildtype C57BL/6 mice, AAV5-GfaABC1d-GCaMP6f was injected into S1.

### Calcium imaging processing and analysis

Images of SR101-stained astrocytes were used to outline individual astrocyte territories. Selected astrocytes had to be in the focal plane with a fully visualized soma and arborization, not cut off at the edge of the image, or overlapped with vasculature. Images of SR101-stained astrocytes were used to outline individual astrocyte territories using a custom MATLAB program (Calsee: https://www.araquelab.com/code/). Calsee identifies astrocyte structure using a strict thresholding of SR101-positive pixels to form a conservative estimate of astrocyte morphology. Astrocyte territories were further segmented into soma and arborization regions of interest (Supplementary Fig. 8). Individual astrocytes were brought to polar coordinates centered at their soma, which was outlined as the radial region with limits defined by 50% of structural fluorescence. Arborization of astrocyte processes was defined as the adjacent remaining radial territory (i.e., outside the soma) whose fluorescence was >0.25 times the standard deviation over the median of a circle of fluorescence, and represents the ensemble fluorescence of astrocyte processes. Arborization of astrocyte processes were then discretized into a grid of 3 µm × 3 µm square regions, and each of these regions were defined as a microdomain. These structurally-identified regions of interest (i.e., soma, arborization and microdomains) were then used to quantify calcium activity from the simultaneously recorded green channel. Event detection of calcium fluorescence was determined when the amplitude of response was >3 times the standard deviation of the average baseline amplitude.

Calcium responses of astrocyte arborizations and somas to variable sensory stimuli were fitted to the following general sigmoid function:

1 $$R( x ) = \frac{{r_{{\mathrm{max}}}}}{{1 + {\mathrm{e}}^{ - r_{{\mathrm{slope}}}(x - r_{50})}}}$$

where *R*(*x*) was the astrocytic arborization and soma responses, *r* was duration (*D*), frequency (*F*), and intensity (*I*) of the stimulus; *D*_max_, *F*_max_, and *I*_max_ were the maximum responses for the respective stimulus parameter; *D*_slope_, *F*_slope_, and *I*_slope_ were the respective slopes, which are considered as indicative of the astrocyte discrimination of the sensory stimuli, i.e., how the astrocyte activity response changes as the stimulus changes; and *D*_50_, *F*_50_, and *I*_50_ were the stimulus parameter values at which astrocyte responses were 50% of the maximum values, which was considered as indicative of astrocyte sensitivity of sensory stimuli.

### Electrophysiology processing and analysis

Electrophysiology signals were lowpass filtered below 100 Hz using a lowpass FIR filter to restrict signal to fluctuations of the local field potential. Frequency content was extracted from a spectrogram created using a hamming window of length 4096 frames. This spectrogram was converted into decibels and the temporal frequency dynamics were smoothed using Welch’s method. Average power of different frequency bands over time was obtained by averaging frequency content over their respective bands. Spectral analysis was performed on the frequency bands of low (0–1 Hz), delta (0–4 Hz), theta (4–7 Hz), alpha (8–12 Hz), beta (13–30 Hz), and gamma (30–50 Hz). Custom MATLAB code can be found at https://www.araquelab.com/code/.

Power spectrum range of neuronal network activity evoked by variable sensory stimuli were fit to a specialized sigmoid function:

2 $$R( x ) = \frac{{r_{{\mathrm{max}}}}}{{1 + {\mathrm{e}}^{ - r_{{\mathrm{slope}}}(x - r_{50})}}} + r_{{\mathrm{min}}}$$

where *R*(*x*) was the cortical power spectrum responses, *r* was duration (*D*), frequency (*F*), and intensity (*I*) of the stimulus; *D*_max_, *F*_max_, and *I*_max_ were the response maximum values over *r*_min_ (i.e., the minimum value of the respective stimulus parameter); *D*_slope_, *F*_slope_ and *I*_slope_ were the respective slopes; and *D*_50_, *F*_50_, and *I*_50_ were the stimulus parameter values at which cortical power spectrum responses were 50% of the maximum values.

### Immunohistochemistry

Mice were perfused with paraformaldehyde to fix the brain. Brains were extracted and placed in sucrose overnight. Fixed brains were cut to 50 µm slices on a black vibratome. Next, slices were stained for GFAP (rabbit anti-GFAP; Sigma; 1:500) and NeuN (mouse anti-NeuN; Millipore; 1:500) to image astrocytes and neurons, respectively, with mCherry.

### Statistical testing

Astrocyte calcium quantifications were averaged over all astrocytes of a single video and these values were used in statistical testing. Paired and unpaired two-tailed student *t*-tests were performed with *α* = 0.05 against the null hypothesis that no difference exists between the two groups. Correlations were confirmed using a student’s *t*-test against the null hypothesis that no correlation exist. To test the stimulus-dependence of a stimulus response curve, 1-way ANOVAs were performed with *α* = 0.05 against the null hypothesis that no dependence exists. In the comparisons of two groups’ response curves a 2-way ANOVA was performed with *α* = 0.05 against the additional null hypotheses that that the two groups are the same and no interaction exists. In some examinations following a significant 1-way ANOVA, multiple comparison testing was performed using Tukey’s range test using *α* = 0.05 against the null hypothesis that no samples are different from each other.

### Reporting summary

Further information on research design is available in the Nature Research Reporting Summary linked to this article.

## Supplementary information

Supplementary Information

Peer Review File

Reporting Summary

## Data Availability

Source data are provided as a Source Data file. The datasets generated during and/or analyzed during the current study are available at https://gin.g-node.org/justinlines/Lines_et_al_2020.

## Source data

Source Data

## Code availability

The code used in this study can be found at www.araquelab.com/code Source data are provided with this paper.
