# Supplementary material for: Astrocytes modulate sensory-evoked neuronal network activity
Source: Nat Commun. 2020 Jul 23;11:3689. doi: 10.1038/s41467-020-17536-3 (PMC7378834; doi:10.1038/s41467-020-17536-3)
Supplement: Supplementary file 1 — Supplementary Information [file 41467_2020_17536_MOESM1_ESM.pdf]

Supplementary Information for:

"Astrocytes modulate sensory-evoked neuronal network activity"

Lines et al.

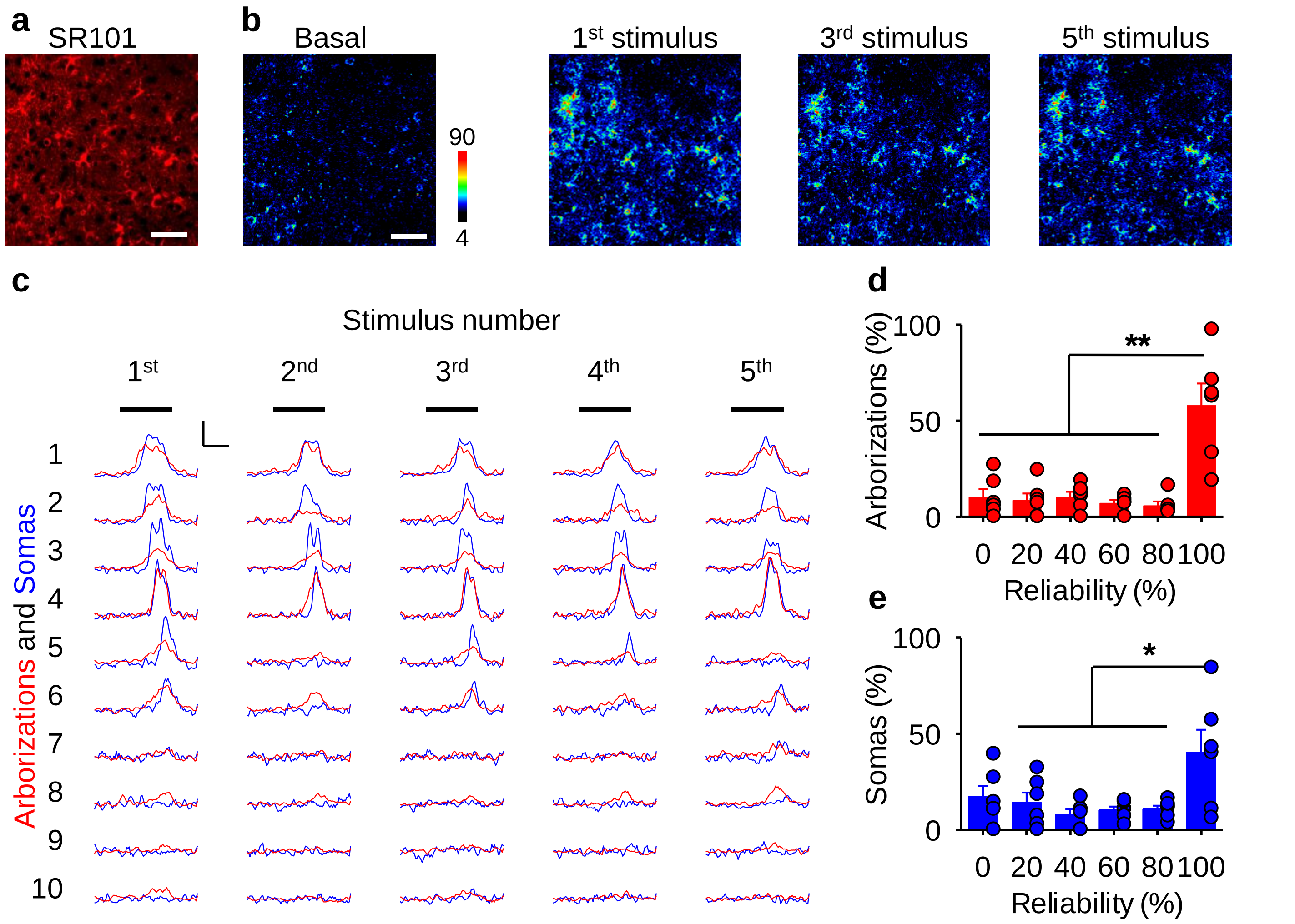

Supplementary Fig. 1 Astrocyte population responses to sensory stimulation are reliable. **a** SR101 staining. Scale bar = 50 µm. **b** Pseudocolor Ca^{2+} image of basal during the 1st, 3rd and 5th hind-paw stimulus. Scale bar = 50 µm. **c** Soma (blue) and arborization (red) Ca^{2+} traces in response to 5 repeated stimuli. Scale = F/F_o, 10 s. Images and traces are representative of data obtained independently across 3 mice. **d** Astrocyte arborizations reliability, quantified as the proportion of successful responses to 5 stimuli (1-way ANOVA: $p = 4.02e^{-7}$; Tukey HSD: $p < 1.1e^{-5}$; n = 6 populations, 3 animals). **e** Astrocyte somas reliability (1-way ANOVA: $p = 0.007$; Tukey HSD: $p < 0.047$; n = 6 populations, 3 animals). Means ± SEM, '*': $p < 0.05$ and '**': $p < 0.01$ Tukey HSD after significant 1-way ANOVA. Source data are provided as a Source Data file.

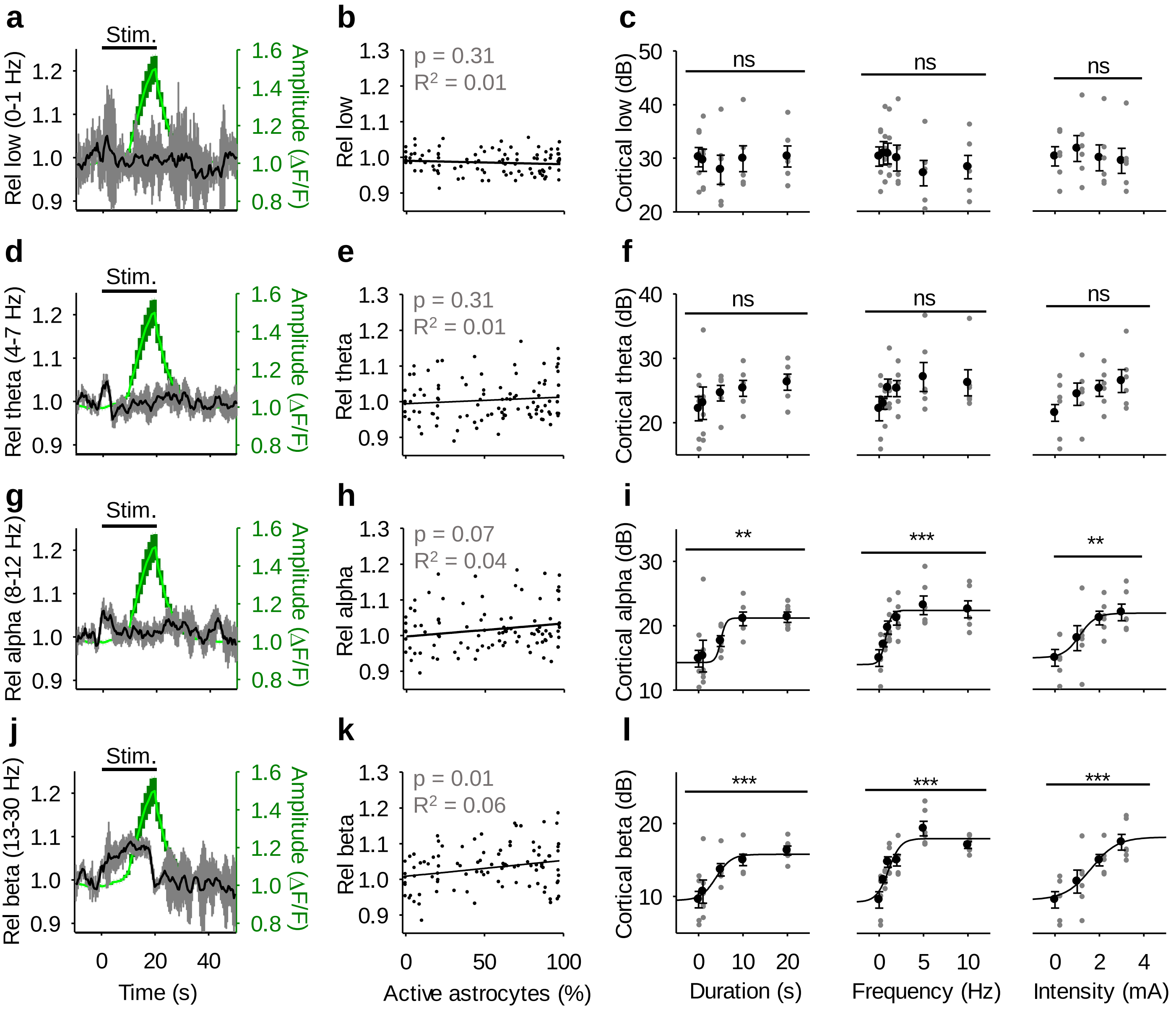

Supplementary Fig. 2 Cortical astrocyte activity co-occurs with increases in ECoG signatures of neuronal network activity during sensory stimulation. **a** Astrocyte Ca^{2+} levels (green) and relative low ECoG frequencies (0-1 Hz; black) vs. time. Lines and shadows represent mean and SEM, respectively, as in other panels. **b** Relative cortical low ECoG frequencies vs. percentage of active astrocytes. Data were fitted to a linear regression with p and R^2 values as indicated. **c** Steady-state cortical low ECoG frequencies vs. the duration (*D*; left; p = 0.93; 1-way ANOVA; n = 6 animals), frequency (*F*; center; p = 0.78) and intensity (*I*; right; p = 0.90) of the sensory stimulation. **d** - **f** as a - c but for cortical theta frequency (4-7 Hz) (duration: p = 0.43, frequency: p = 0.28, intensity: p = 0.33). **g** - **i** as a - c for cortical alpha frequency (8-12 Hz) (duration: p = 0.006, frequency: p = 0.00007, intensity: p = 0.009). **j** - **l** as a - c for cortical beta frequency (13-30 Hz) (duration: p = 0.0004, frequency: p = $1.5e^{-8}$, intensity: p = 0.0007). In i and l, data were fit to the sigmoid function in equation [2] (Methods; see Supplementary Table 1 for fitting parameters). Mean ± SEM. ‘ns’: p > 0.05, ‘**’: p < 0.01, and ‘***’: p < 0.001 using 1-way ANOVA. Source data are provided as a Source Data file.

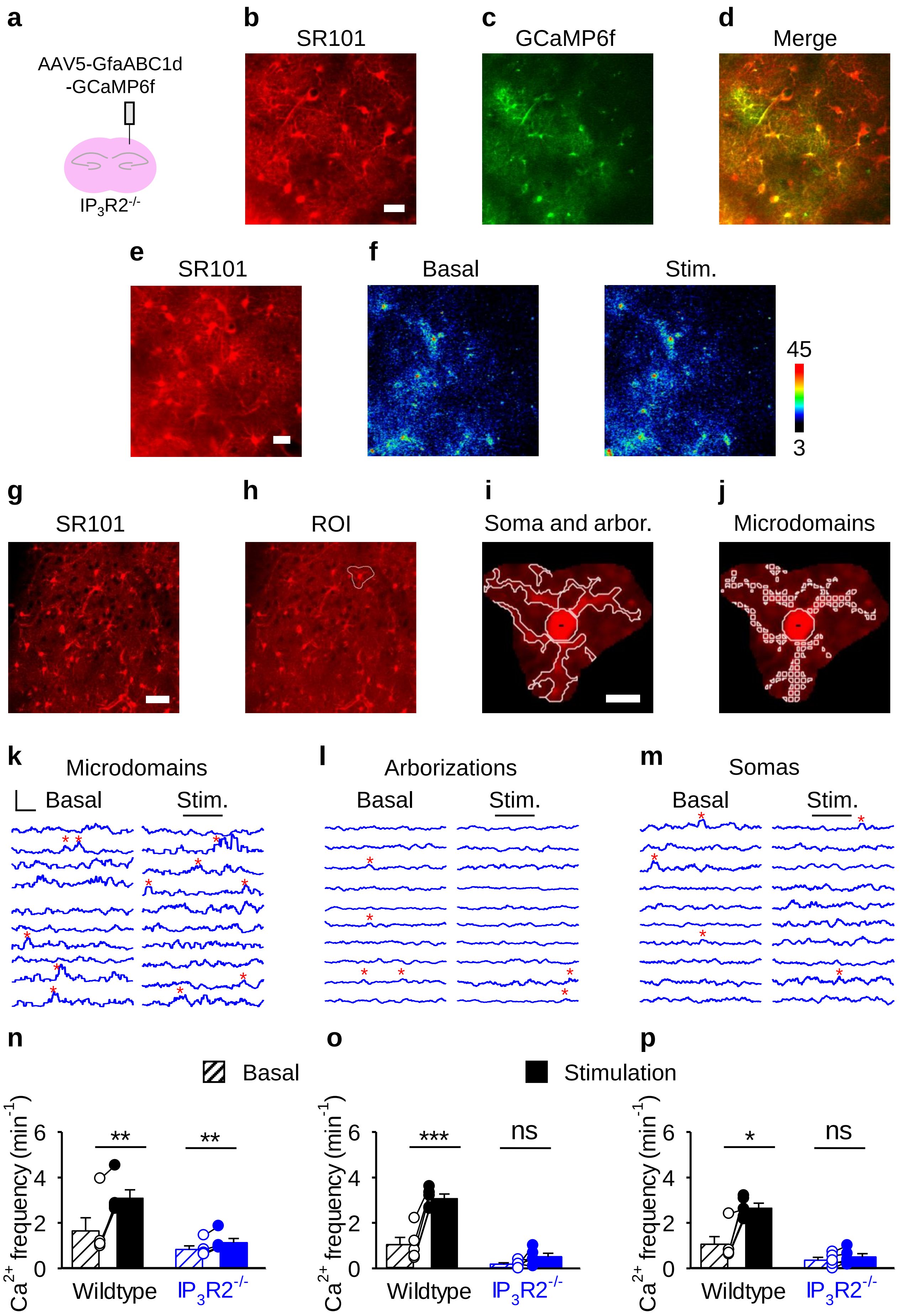
a
AAV5-GfaABC1d
-GCaMP6f
IP3R2-/-
b
SR101
c
GCaMP6f
d
Merge
e
SR101
f
Basal
Stim.
45
3
g
SR101
h
ROI
i
Soma and arbor.
j
Microdomains
k
Microdomains
Basal
Stim.
l
Arborizations
Basal
Stim.
m
Somas
Basal
Stim.
n
o
p
Basal
Stimulation
Ca2+ frequency (min-1)
6
4
2
0
**
**
***
ns
*
ns
Wildtype
IP3R2-/-

Supplementary Fig. 3 Astrocyte calcium signaling is impaired in $IP_3R2^{-/-}$ mice. **a** Scheme of viral injections (AAV5-GfaABC1d-GCaMP6f) into the S1 cortex of $IP_3R2^{-/-}$ mice. **b** SR101 stained astrocytes. Scale bar = 20 µm. **c** GCaMP6f virus infected astrocytes. **d** Merge of SR101 and GCaMP6f images. **e** SR101 stained astrocytes. Scale bar = 20 µm. **f** Pseudocolor Ca^{2+} images during basal and sensory stimulation. **g** SR101 of astrocytes. Scale bar = 50 µm. **h** Example of selected astrocyte (white outline). **i** Soma and arborization segmentation obtained with Calsee program. Scale bar = 10 µm. **j** Discretized microdomains obtained with Calsee program. **k** Calcium traces of subcellular microdomains during basal and sensory stimulation in $IP_3R2^{-/-}$ mice. Red asterisks indicate detected Ca^{2+} events. Scale = F/F_o, 10 s. **l** as k, but for astrocyte arborizations. **m** as k, but for astrocyte somas. **n** Frequency of Ca^{2+} events during basal (hashed) and stimulated (filled) in microdomains in wildtype (black; p = 0.003; paired t-test; n = 5 imaging planes from 2 animals) and $IP_3R2^{-/-}$ (blue; p = 0.0013; paired t-test; n = 5 imaging planes from 2 animals) mice. **o** as n, but for astrocyte arborizations (WT: p = 0.0002; KO: p = 0.15). **p** as n, but for astrocyte somas (WT: p = 0.01; KO: p = 0.17). Mean ± SEM. ‘ns’: p > 0.05, ‘*’: p < 0.05, ‘**’: p < 0.01, and ‘***’: p < 0.001 using two-sided paired student t-test. Images and traces are representative of data obtained independently across 2 mice. Source data are provided as a Source Data file.

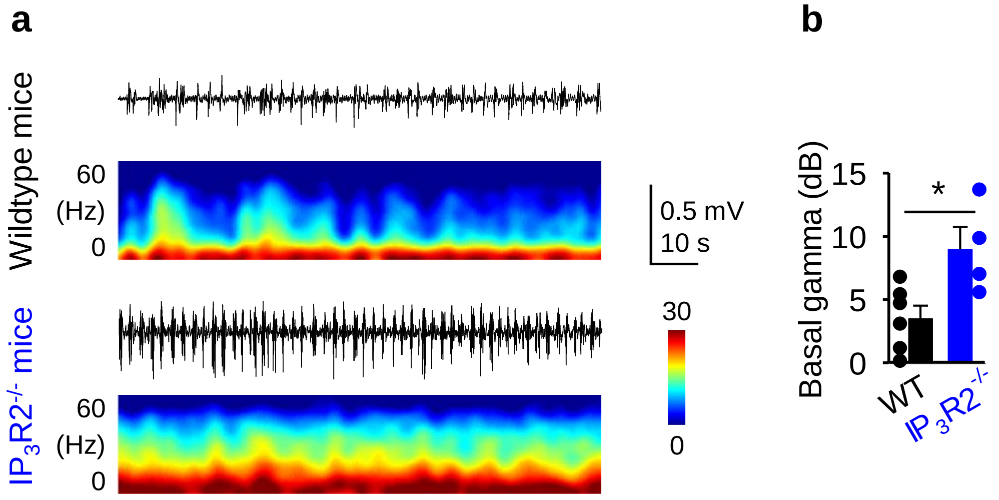

Supplemental Fig. 4 Basal cortical gamma is heightened in *$IP_3R2^{-/-}$* mice. **a** Basal extracellular recordings and spectrograms from wildtype (top) and *$IP_3R2^{-/-}$* (bottom) mice. Traces are representative of data from independent experiments from 4 mice. **b** Basal gamma activity from wildtype (black) and *$IP_3R2^{-/-}$* (blue) mice ($p = 0.02$; two sample t-test; $n = 6$ WT and 4 KO animals). Mean ± SEM. '*': $p < 0.05$ using two-tailed student t-test. Source data are provided as a Source Data file.

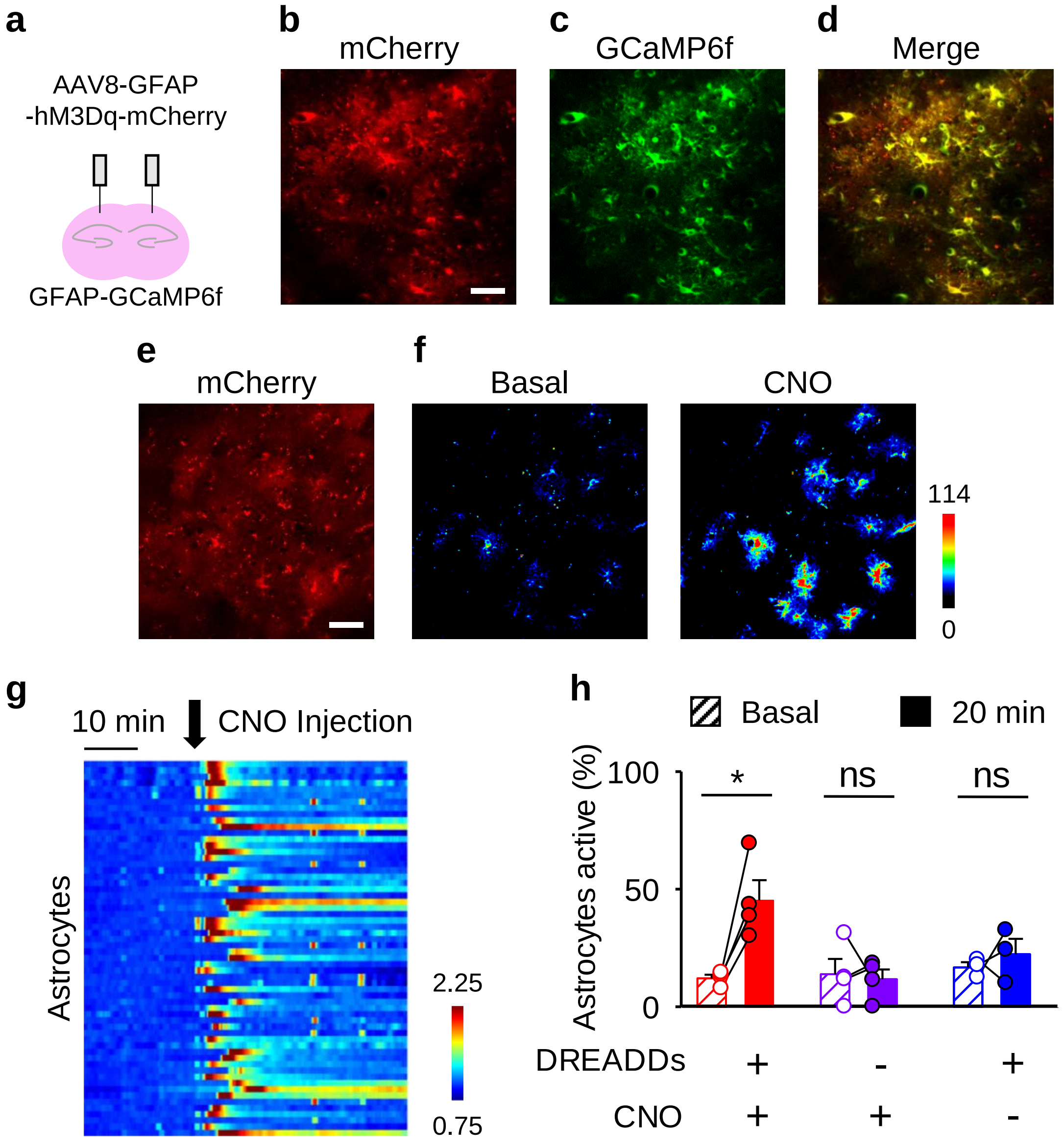

Supplementary Fig. 5 Intraperitoneal injection of Clozopine N-Oxide activates DREADDs expressing astrocytes in vivo. **a** Scheme of bilateral viral injections of either DREADDs (AAV8-GFAP-hM3Dq-mCherry) or control (AAV8-GFAP-mCherry) viral vectors. **b** mCherry expression in S1. Scale bar = 50 µm. **c** GCaMP6f expression in S1. **d** Merge of b and c. **e** mCherry expression in S1. Scale bar = 50 µm. **f** Pseudocolor Ca^{2+} images of astrocyte activity during basal and after CNO injection. Example images are representative of independent data gathered from 4 mice. **g** A total of 60 astrocytes responding to DREADDs activation from 4 different mice. **h** Active astrocytes before (hashed) and 20 minutes after (filled) injections of astrocytes expressing DREADDs with CNO (red: p = 0.02; paired t-test; n = 4 mice), mCherry with CNO (purple; p = 0.58; n = 4 mice), and DREADDs with saline (blue; p = 0.73; n = 3 mice). Mean ± SEM. ‘ns’: p > 0.05 and ‘*’: p < 0.05 using two-sided paired student t-test. Source data are provided as a Source Data file.

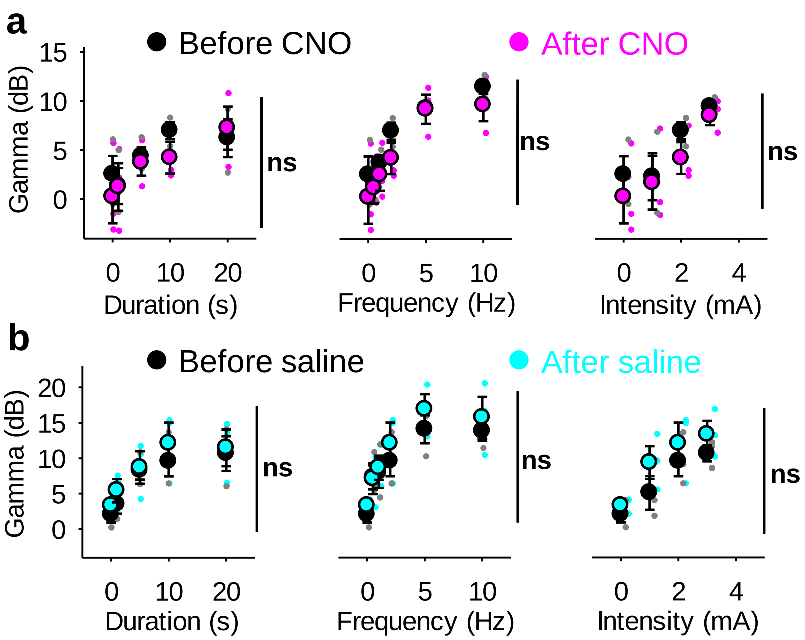

Supplementary Fig. 6 Introducing CNO or surgery does not modulate cortical neuronal network response to sensory stimulation. **a** Cortical gamma in response to sensory stimulation before (black) and after (magenta) intraperitoneal injection of CNO into control virus infected animals (2-way ANOVAs for CNO: duration: $p = 0.43$; frequency: $p = 0.10$; intensity: $p = 0.24$; $n = 3$ animals). **b** Cortical gamma in response to sensory stimulation before (black) and after (cyan) intraperitoneal injection of saline into DREADDs infected animals (2-way ANOVAs for saline: duration: $p = 0.27$; frequency: $p = 0.21$; intensity: $p = 0.07$; $n = 3$ animals). Mean ± SEM. ‘ns’: $p > 0.05$ for 2-way ANOVA. Source data are provided as a Source Data file.

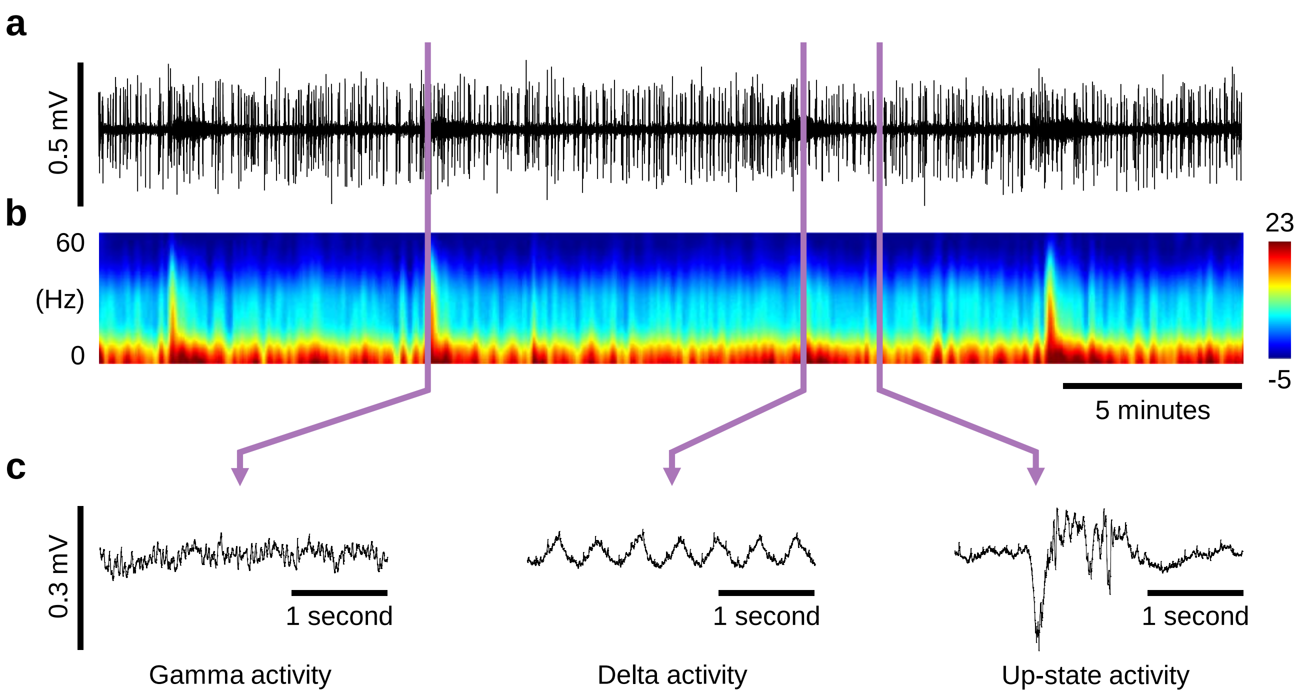

Supplementary Fig. 7 Stability of ECoG signatures in the primary somatosensory cortex in vivo. **a** Raw ECoG signal monitoring basal activity in S1 ($-1_{a\text{-}p}$, $1.5_{m\text{-}l}$). **b** Spectrogram of frequency content in the ECoG. **c** Expanded traces showing distinct cortical activity in basal recordings: gamma activity, delta activity, and Up-state activity.

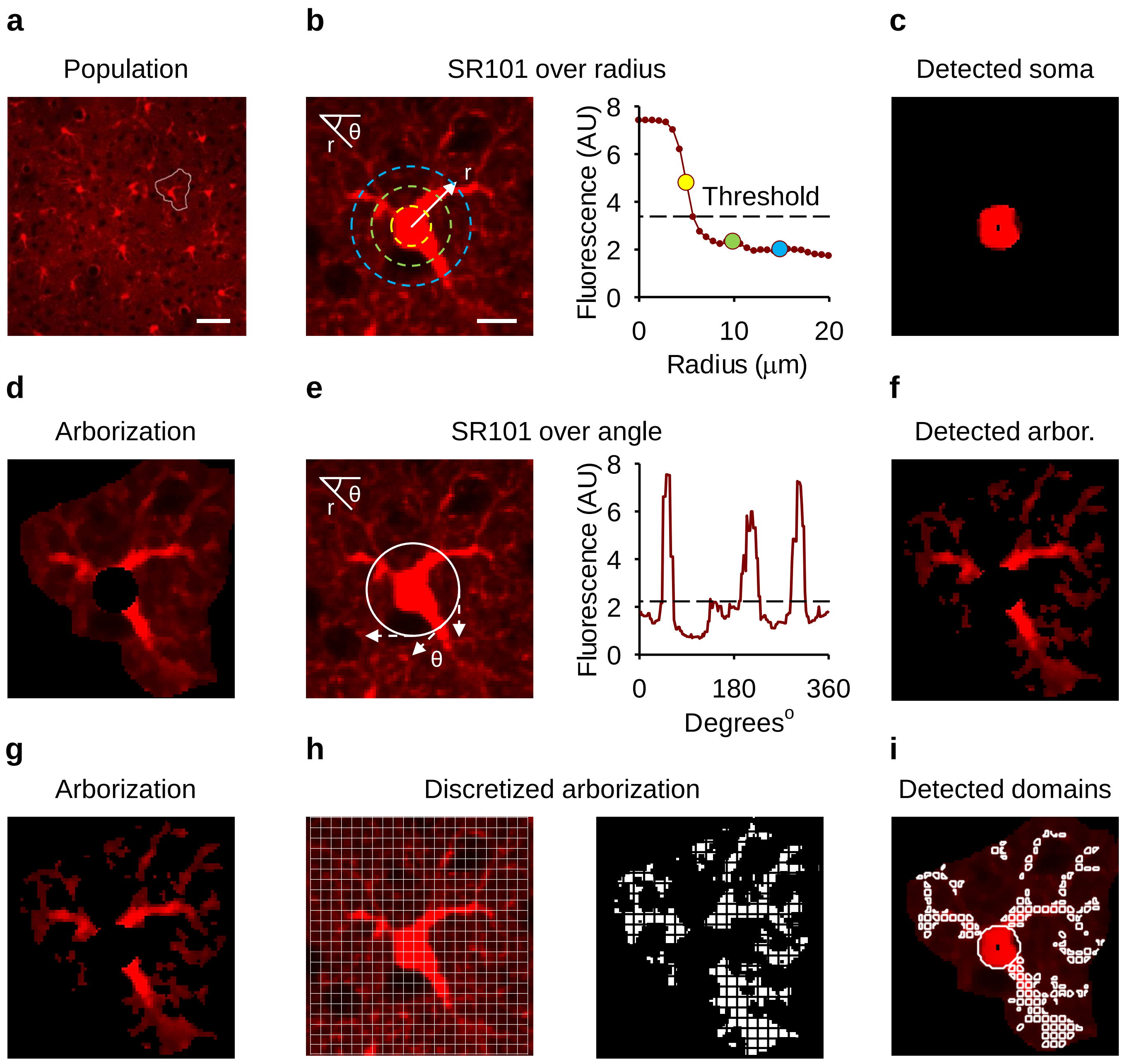

Supplementary Fig. 8 Semi-automatic method for the segmentation of SR101 astrocyte morphology. **a** SR101 stained astrocyte population with an outlined cell (white outline). Scale bar = 50 µm. **b** Selected astrocyte placed in polar coordinates with radial distance (**r**) and polar angle (**θ**) to assess the radius of the soma (left; scale bar = 10 µm) and the average fluorescence of circles centered on the astrocyte soma as radius is extended outward (right) with the threshold = 50% reduction in fluorescence (dashed line). Yellow, green and blue dots on the curve correspond to the average fluorescence of the respectively colored circles over the astrocyte. **c** Resulting image of the isolated soma. **d** Astrocyte in b sans soma. **e** Selected astrocyte placed in polar coordinates with overlaid circle to assess structural fluorescence and the fluorescence vs. angle (right) with the threshold = 0.25 times the standard deviation + median fluorescence (dashed line). Note the neuron at 90º corresponding to a low SR101 fluorescence. **f** and **g** Resulting image of the isolated arborization. **h** Grid of 3 µm x 3 µm squares over the astrocyte (left) and the discretized segmented arborization showing discrete regions of interest (ROI) (right). **i** Resulting ROIs labeling the microdomains and soma. This example is representative of over 1000 ROIs.

	Low (0-1Hz)	theta (4-7 Hz)	alpha (8-12Hz)	beta (13-30Hz)
D_{min} (dB)	n.a.	n.a.	14.4	9.5
D_{max} (dB)	n.a.	n.a.	21.2	15.8
D_{slope} (s^{-1})	n.a.	n.a.	1.7	0.6
D_{50} (s)	n.a.	n.a.	5.0	4.0
F_{min} (dB)	n.a.	n.a.	14.4	9.5
F_{max} (dB)	n.a.	n.a.	22.3	18.0
F_{slope} (Hz^{-1}	n.a.	n.a.	3.0	1.5
F_{50} (Hz)	n.a.	n.a.	0.8	1.1
I_{min} (dB)	n.a.	n.a.	14.4	9.5
I_{max} (dB)	n.a.	n.a.	21.8	18.2
I_{slope} (mA^{-1}	n.a.	n.a.	2.8	1.7
I_{50} (mA)	n.a.	n.a.	1.1	1.6

Supplementary Table 1 Fitting parameters of ECoG frequency activity to eq. [2].

	Wildtype	DREADDs	$IP_3R2^{-/-}$
D_{min} (dB)	3.2	1.0	8.4
D_{max} (dB)	9.4	5.6	10.8
D_{slope} (s^{-1})	0.5	1.9	1.6
D_{50} (s)	3.8	4.6	4.9
F_{min} (dB)	3.2	1.0	8.4
F_{max} (dB)	12.0	6.9	14.6
F_{slope} (Hz^{-1})	1.4	2.0	1.0
F_{50} (Hz)	1.1	0.9	2.7
I_{min} (dB)	3.2	1.0	8.4
I_{max} (dB)	12.8	7.2	13.3
I_{slope} (mA^{-1})	1.4	2.5	1.1
I_{50} (mA)	1.7	1.2	2.0

Supplementary Table 2 Fitting parameters to eq. [2] of ECoG gamma activity in wildtype mice, CNO activation of astrocytes expressing DREADDs, and IP3R2-/- mice.
