# Supplementary material for: Astrocytes modulate sensory-evoked neuronal network activity
Source: Nat Commun. 2020 Jul 23;11:3689. doi: 10.1038/s41467-020-17536-3 (PMC7378834; doi:10.1038/s41467-020-17536-3)
Supplement: Supplementary file 2 — Peer Review File [file 41467_2020_17536_MOESM2_ESM.docx]

Peer Review Information

**Journal:** Nature Communications

**Manuscript title:** Astrocytes modulate sensory-evoked neuronal network activity

**Corresponding author name(s):** Dr Alfonso Araque

**Editorial notes:**

**Reviewer comments & decisions:**

**Reviewer comments, first version:**

Reviewers' comments:

Reviewer #1 (Remarks to the Author):

This is an interesting and generally well-performed study, from an internationally established, highly productive group. It deals with an important and timely issue, specific functional interactions between astroglial and neuronal networks in response to sensory stimuli. The authors combine astrocyte calcium imaging in vivo with ECoG recordings, which is an important methodological advance in the field. They find correlations between high-, but not low-frequency ECoG activities and astroglial calcium signals. Interestingly, they detect only a relatively minor effect of knocking out astroglial IP3R2 (major calcium cascade) on the stimulus-evoked ECoG response. They do observe a causal effect of DREAD-based astroglial activation on the neuronal response to sensory stimulation. The authors confidently use up-to-date, state-of-the-art techniques and follow a straightforward flow of logic. They might want to address some of issues highlighted below.

1. As a general note, it might be important to explain further the conceptual advance distinguishing the present work from other studies that reported the impact of astroglial manipulations on neural activity or behavior.

2. Abstract. The fact that astrocytes are 'important cells' scarcely needs any proof. One can readily assume that all brain cells are important, one way or the other. The authors are encouraged to re-phrase this, perhaps by emphasizing the aspects of novelty and importance.

3. ECoG: It would seem important to expand further on the quality control and stability criteria for ECoG recordings, to characterize baseline requirements, filtering parameters, the baseline-control comparison criteria, etc. The Fig. 1c trace depicts a dramatic drop in the (low-frequency) ECoG activity, which does not seem to get reflected in the subsequent analyses. The Fig. 4c ECoG trace looks altogether different: transient bursts during the baseline period are reminiscent of interictal spikes, which if true would be worrying, in the context.

4. Figs. 1-2. This nice piece of data leaves an impression of a novel observation whereas responses of astroglia to sensory stimuli have previously been reported and described in some detail (e.g., Wang et al, Nat Neuro 2006; Zhao et al , Mol Brain 2012; Reynolds et al, Neurosci Lett 2018). The authors are encouraged to acknowledge previous work and explain further the novelty and impact of their own observations.

5. Fig. 4. These data appear to correspond to a saturating sensory stimulus (2 mA, 5 Hz) applied for 60 s. In such cases, the brain response is likely to involve various time-dependent desensitization and adaptation processes. It would seem important to explore the possible concomitants involved.

6. Fig. 4g: When normalized to the baseline (the method adopted throughout the study), these data show no difference in gamma-activity between WT and IP3R2 KO animals. This needs to be explained.

7. Fig. 5f-g: The data seem to show an enormous increase in baseline ECoG activity in the IP3R2 KO mice compared to WT. It would be important to know how 'healthy' this ECoG is, and whether Fig. 5f data are consistent with Fig. 4g data, in this context.

8. The authors are encouraged to discuss the possible network organizing principles and signaling cascades that could explain the reported phenomena.


Reviewer #3 (Remarks to the Author):

Lines and colleagues explore the role of astrocytic Ca2+ signalling in the processing of sensory stimuli. They start by performing Ca2+ imaging in astrocytes of the somatosensory cortex in anesthetized mice while stimulating the hind paw electrically. They first demonstrate that somatic regions of the astrocytes and their arborization respond reliably and that the responses increase with stimulation duration, frequency and intensity. The simultaneously recorded ECoG displays a similar dependency on the stimulation in the alpha to gamma bands. Interestingly, the astrocyte Ca2+ signals follow the ECoG gamma activity with a delay and terminate before the end of the stimulation and the gamma activity increase. This relationship appears to be altered in IP3R2 knockout animals. Experimental stimulation of astrocyte Gq signalling using DREADDS dampens the gamma activity increase in response to stimulation. These experiments clearly show that astrocytic Ca2+ signalling changes after sensory stimulation are correlated with ECoG activity pattern changes and that manipulating astrocytic Ca2+ signalling affects the gamma response. Overall, this is a very interesting and timely study with elegant experiments and with the important and new result that astrocytic Ca2+ signalling shapes neuronal cortical responses triggered by activation of somatosensory inputs.

Major comments:

1) The analysis of imaging data is incompletely described. The authors analyse Ca2+ signals in automatically defined ROIs of 3 x 3 µm2. The illustrated ROIs in Fig. 1b and f do not fill the entire territory of the cell. Why is that? Are only active ROIs shown in these examples? In Fig. 1h, Ca2+ traces from microdomains are shown (pink). The authors need to explain and illustrate with examples, how microdomains, arborization and somatic ROIs were defined. Although the authors show microdomain events (Fig. 1h) these never appear be an analysed in depth. Or are they later pooled with all other transients to calculate the active percentages?

2) To fully interpret the ECoG data from IP3R2 knockout mice and DREADD experiments, it is important to know how these manipulations affect the Ca2+ signals under baseline condition and during sensory stimulation. Doing both sets of experiments might not be feasible. It is important however for the IP3R2 knockouts because there is a significant dampening of gamma later during the stimulation (Fig. 4g) and a significant increase of gamma activity during the baseline period (Fig. 5f). Given the variable results in the literature regarding the dependence of (microdomain) Ca2+ signals in astrocytes on IP3R2 it is important to include the microdomain category in the analysis throughout.

3) The experiments presented in figures 4 and 5 are very interesting and insightful. The baseline gamma activity is higher in IP3R2 knockouts (Fig. 5f) and this seems to partially occlude further increases during stimulation (Fig. 5g). The authors should compare the gamma baseline levels and the relative gamma activity increases during stimulation between control and DREADD experiments and experiments using IP3R2 knockouts. The control data in Fig. 5d-e (‘before CNO’) and Fig. 5f-g (‘wildtype mice’) look identical. If correct, this should be made clear in the legend and labelling.

4) There are a few questions that could be discussed: Why is there a delay of ~ 10 seconds between the onset of the Ca2+ signals and the stimulation? Why does the Ca2+ signal terminate well before the end the stimulation although the effect on gamma is persisting? What cellular mechanisms drive the Ca2+ signals how do they alter gamma activity?


Minor points

1) SR101 has been shown to label also oligodendrocytes and to increase neuronal excitability. This should be discussed.

2) It would be helpful to mention in the legend of Fig. 2 what D, F and I refer to. Triple asterisks are shown but not explain in the legend. The data is obtained from 11 ‘populations’. How many independent preparations/mice does that correspond to? Have the authors analysed the delay between the onset of stimulation and the astrocytic Ca2+ signal? I would expect that increasing the stimulation frequency or intensity reduces the delay.

3) In the figures 3e and 3f, the values for R2 are very low. This needs to be explained or discussed. Would splitting the data into low and high activity ranges and comparison of the relative delta/gamma activities between those groups give a clearer picture? According to Fig. 2, long/strong/frequent stimuli are associated with a high number of active astrocytes, which is according to Fig. 3e associated with low relative delta activity. What is the explanation that this is not seen in Fig. 3g?

4) The demonstration of selective DREADD (mCherry) expression in astrocytes in Fig. 5a is not ideal. Although it clearly shows that neuronal somas are negative for mCherry, it is hard to tell how well the diffuse mCherry signal colocalizes with astrocytic structures. Have the authors quantified the specificity?

5) Please specify the frequency bands also in the material and methods section.

**Author rebuttal, first version:**

(Reviewer comments are in *blue italics*; sentences included in the manuscript text are in **bold**).

*Reviewer #1:*

*This is an interesting and generally well-performed study, from an internationally established, highly productive group. It deals with an important and timely issue, specific functional interactions between astroglial and neuronal networks in response to sensory stimuli. The authors combine astrocyte calcium imaging in vivo with ECoG recordings, which is an important methodological advance in the field. They find correlations between high-, but not low-frequency ECoG activities and astroglial calcium signals. Interestingly, they detect only a relatively minor effect of knocking out astroglial IP3R2 (major calcium cascade) on the stimulus-evoked ECoG response. They do observe a causal effect of DREAD-based astroglial activation on the neuronal response to sensory stimulation. The authors confidently use up-to-date, state-of-the-art techniques and follow a straightforward flow of logic. They might want to address some of issues highlighted below.*

We thank the reviewer for the very positive assessment of the study and the helpful comments and suggestions that have helped to improve the manuscript.

1. *As a general note, it might be important to explain further the conceptual advance distinguishing the present work from other studies that reported the impact of astroglial manipulations on neural activity or behavior.*

Following the reviewer´s suggestion, we have added the following paragraph in the Introduction: “**Therefore, while previous studies have shown the ability of cortical astrocytes to respond to sensory stimulation and contribute to cortical neuronal network states ^12-15,21,^ we here extend these observations by providing the quantification of sensory-evoked astrocyte responsiveness and the impact on sensory-evoked cortical neuronal network activity, quantitatively determining the parameters that define the relationship between sensory stimuli and cortical astrocyte-neuronal network responses.**” (P. 3, l. 66).

2. *Abstract. The fact that astrocytes are 'important cells' scarcely needs any proof. One can readily assume that all brain cells are important, one way or the other. The authors are encouraged to re­phrase this, perhaps by emphasizing the aspects of novelty and importance.*

Following the reviewer´s suggestion, we have modified the sentence: “**In contrast with the accepted paradigm that neurons principally mediate brain function, astrocytes are emerging as cells with important neuromodulatory actions in brain physiology.**” (P. 2, l. 29).

3. *ECoG: It would seem important to expand further on the quality control and stability criteria for ECoG recordings, to characterize baseline requirements, filtering parameters, the baseline-control comparison criteria, etc. The Fig. 1c trace depicts a dramatic drop in the (low-frequency) ECoG activity, which does not seem to get reflected in the subsequent analyses. The Fig. 4c ECoG trace looks altogether different: transient bursts during the baseline period are reminiscent of interictal spikes, which if true would be worrying, in the context.*

We thank the reviewer for rising the comments that allowed us to clarify the issues. We have expanded the description of electrophysiological experiments under the subheading “In vivo electrophysiology” to address the reviewer´s comments. Specifically:

Regarding quality control and stability criteria for ECoG recordings, we have added the following clarification in Materials and Methods section: “**Before delivering sensory stimulation protocols, electrophysiological recordings were monitored for up to 30 minutes for stability, i.e. main sustained activity in the low frequency range with occasional regular transient activity in the gamma range (Supplementary Fig. 7). The regular transient events were identified as up-states according to their amplitude (0.17 ± 0.05 mV) duration (0.93 ± 0.10 s) and waveform pattern (Supplementary Fig. 7c). The few animals that did not show stable recordings were discarded.**

**To ensure cortical activity was restored to baseline levels following stimulation, 2-3 minutes of interstimulus times were taken before the subsequent stimulation was performed.**” (P. 17, l. 450).

Regarding filtering parameters, etc., we have included the following sentence in Materials and Methods section: “**Electrophysiological recordings were sampled at 10 kHz using an A-M Systems model 3000 ACDC differential amplifier, band pass filtered between 1 – 3000 Hz, and digitized using an Axon Digidata 1550A acquisition system connected to a PC and monitored with AxoScope software (pCLAMP).**” (P. 17, l. 447).

Regarding comment to Fig. 1c (actually Fig. 3c), we agree with the reviewer that the selected case was not a good representative example of the average phenomenon shown in Fig. 3d. We have therefore substituted the illustrative example for a more representative one (**new panel c in Fig. 3**).

Regarding comment to Fig. 4c, we agree with the reviewer that the transient bursts during the baseline period could be mistakenly taken as interictal spikes due to the compressed trace shown. However, the expanded trace displayed in the **new Supplementary Fig. 7** shows that the events are actually regular transient up-states, as identified by their amplitude, duration and waveform. Hence, to clarify the issue we have added the **new Supplementary Fig. 7** and included the following sentence in Materials and Methods section “**The regular transient events were identified as up-states according to their amplitude (0.17 ± 0.05 mV) duration (0.93 ± 0.10 s) and waveform pattern (Supplementary Fig. 7c).**” (P. 17, l. 453).

4. *Figs. 1-2. This nice piece of data leaves an impression of a novel observation whereas responses of astroglia to sensory stimuli have previously been reported and described in some detail (e.g., Wang et al, Nat Neuro 2006; Zhao et al , Mol Brain 2012; Reynolds et al, Neurosci Lett 2018). The authors are encouraged to acknowledge previous work and explain further the novelty and impact of their own observations.*

We thank the reviewer for the praising comment and the suggestion. As indicated in the response to comment #1, we have included paragraphs in the Introduction to acknowledge previous reports and to explicitly state the novelty of our study: “**Therefore, while previous studies have shown the ability of cortical astrocytes to respond to sensory stimulation and contribute to cortical neuronal network states ^12-15,21,^ we here extend these observations by providing the quantification of sensory-evoked astrocyte responsiveness and the impact on sensory-evoked cortical neuronal network activity, quantitatively determining the parameters that define the relationship between sensory stimuli and cortical astrocyte-neuronal network responses.**” (P. 3, l. 66), and in the Discussion “**While previous studies have reported on cortical astrocytes responses to somatosensory stimulation and astrocyte influence on cortical neuronal network states 12-15,21, present results provide the first quantification of the sensory-evoked astrocyte responsiveness and the impact on sensory-evoked cortical neuronal network activity.**” (P. 11, l. 274).

5. *Fig. 4. These data appear to correspond to a saturating sensory stimulus (2 mA, 5 Hz) applied for 60 s. In such cases, the brain response is likely to involve various time-dependent desensitization and adaptation processes. It would seem important to explore the possible concomitants involved.* We fully agree with the reviewer. Indeed, the stimulation delivered to obtain the results presented in Fig. 4, which was selected to optimally activate astrocytes, may likely involve additional time-dependent desensitization and adaptation processes. However, after determining the stimulus-dependent responses in previous figures, we designed this specific experiment to clearly reveal the astrocyte regulatory feedback. We agree that studying the involvement of potential additional processes is certainly an important issue that would require further complex studies beyond the scope of the present work. To clarify the issue, we have added the following paragraph in the Discussion section: “**After determining the stimulus-dependency of the astrocyte and neuronal network responses (Figs.**

**1-3), a strong long-lasting stimulus was designed to test the regulatory feedback signaling provided by astrocytes (Fig. 4). The results obtained by delivering a more prolonged stimulus also indicate that astrocytes contribute to the cortical sensory adaptation. Indeed, the decline of gamma cortical activity associated with the delayed astrocyte calcium rise and the hysteresis relationship between gamma activity and astrocyte calcium suggests the astrocyte regulation of sustained gamma activity. The responses to this type of stimulation is likely to involve additional time-dependent desensitization and adaptation processes. While further studies are required to determine the involvement of additional neuronal mechanisms, our results support the idea that astrocyte activity regulates the steady-state of the sensory-evoked gamma network activity.**” (P. 12, l. 326)

6. *Fig. 4g: When normalized to the baseline (the method adopted throughout the study), these data*

*show no difference in gamma-activity between WT and IP3R2 KO animals. This needs to be explained.* We thank the reviewer for the insightful comment.

As indicated by the reviewer, we indeed normalized gamma activity to baseline in most cases because the basal levels are relatively homogeneous. However, since basal gamma activity was found to be heightened in IP3R2^-/-^ animals (**new Supplementary Fig. 4**), we believe that normalization of the responses might provide misleading results, i.e., the sensory-evoked gamma activity might appear affected when compared to wildtype mice because the initial enhanced basal activity. Therefore, we used two alternative analysis. First, we have fitted the data presented in Fig. 5 to the equation [2] to obtain the parameters that define the stimulus-dependency of the responses in both wildtype and IP3R2^-/-^ mice. The results are reported in the **new Table 2** and illustrate that the dynamic range of the sensory-evoked gamma activity is reduced in IP3R2^-/-^ mice**.** Furthermore, since we aimed to test whether the astrocyte calcium contributed to the decline of the sensory-evoked gamma activity from the peak to the steady-state, we quantified the steady-state/peak ratio (**new Fig. 4h**). We have added the following paragraph to describe the results: "**IP3R2^-/-^ mice showed heightened basal gamma activity compared to wildtype (3.5 ± 1.0 dB in wildtype vs. 9.0 ± 1.8 dB in IP3R2^-/-^ mice; p < 0.05; n = 4 animals; Supplementary Fig. 4), and, like wildtype mice, responded to sustained sensory stimulation with the initial surge of gamma activity that declined to a persistent steady state (Fig. 4f-g). However, while the gamma showed similar peak values in wildtype and IP3R2^-/-^ mice (14.7 ± 0.7 dB and 16.2 ± 1.1 dB respectively; p = 0.30; n = 5 animals each; Fig. 4h), it reached higher steady-state values in IP3R2^-/-^ mice (steady-state: 9.1 ± 0.3 dB in wildtype vs. 12.2 ± 0.7 dB in IP3R2^-/-^; p < 0.01; Fig. 4h). Moreover, the temporal decline of gamma activity during the sensory stimulation was significantly diminished in IP3R2^-/-^ mice, as evidenced by the steady-state/peak ratios (62.2 ± 3.7 % in wildtype vs. 79.5 ± 1.4 % in IP3R2^-/-^ mice; p < 0.01; Fig. 4h). Taken together, these results show that the dynamic range of the gamma activity is reduced in IP3R2^-/-^ mice, and indicate that astrocyte activity regulates the steady-state of the sensory-evoked gamma network activity.**” (P. 8, l. 200).

7. *Fig. 5f-g: The data seem to show an enormous increase in baseline ECoG activity in the IP3R2 KO mice compared to WT. It would be important to know how 'healthy' this ECoG is, and whether Fig. 5f data are consistent with Fig. 4g data, in this context.*

As indicated in the previous point, basal gamma activity in IP3R2^-/-^ mice was indeed heightened relative to wildtype animals (**new Supplementary Fig. 4**). This alteration is probably due to intrinsic changes occurring in this constitutive transgenic line, rather than “unhealthy” ECoG recordings, because it was observed consistently in different individuals (note the relatively small SEM in data presented in Supplementary Fig. 4b). Technical differences in recordings form wildtype and IP3R2^-/-^ mice can be also ruled out because experiments with both types of mice were performed in a random and intermingled sequence. We have added the following paragraph to clarify the issue and highlight the notable basal gamma activity in IP3R2^-/-^ mice: "**IP3R2^-/-^ mice showed heightened basal gamma activity compared to wildtype (3.5 ± 1.0 dB in wildtype vs. 9.0 ± 1.8 dB in IP3R2^-/-^ mice; p < 0.05; n = 4 animals;**

**Supplementary Fig. 4), and, like wildtype mice, responded to sustained sensory stimulation with the initial surge of gamma activity that declined to a persistent steady state (Fig. 4f-g). However, while the gamma showed similar peak values in wildtype and IP3R2^-/-^ mice (14.7 ± 0.7 dB and 16.2 ± 1.1 dB respectively; p = 0.30; n = 5 animals each; Fig. 4h), it reached higher steady-state values in IP3R2^-/-^ mice (steady-state: 9.1 ± 0.3 dB in wildtype vs. 12.2 ± 0.7 dB in IP3R2^-/-^; p < 0.01; Fig. 4h). Moreover, the temporal decline of gamma activity during the sensory stimulation was significantly diminished in IP3R2^-/-^ mice, as evidenced by the steady-state/peak ratios (62.2 ± 3.7 % in wildtype vs. 79.5 ± 1.4 % in IP3R2^-/-^ mice; p < 0.01; Fig. 4h). Taken together, these results show that the dynamic range of the gamma activity is reduced in IP3R2^-/-^ mice, and indicate that astrocyte activity regulates the steady-state of the sensory-evoked gamma network activity.”** (P. 8, l. 200).

*8. The authors are encouraged to discuss the possible network organizing principles and signaling*

*cascades that could explain the reported phenomena.*

Following the reviewer´s suggestion, we have discussed the issue with the following paragraph included in the Discussion: **“Multiple signaling pathways may be putatively involved in the bidirectional astrocyte-neuron communication underlying the observed results. On the one hand, astrocytes are known to respond with Ca^2+^ elevations to a wide variety of neurotransmitters released at cortical synapses active during the sensory stimulation, including glutamate, GABA, acetylcholine, norepinephrine or dopamine ^9,11,42.^ On the other hand, multiple astrocyte signaling mechanisms may influence neuronal activity ^9,11,^ either through synapse-specific or volume transmission ^43-45.^ Astrocytes activated during sensory stimulation may release transmitters, such as glutamate ^46,47,^ GABA ^48^, or ATP/adenosine ^22,49,^ that may affect neuronal activity as well as excitatory and inhibitory synapses. Additionally, astrocytes can modulate neuronal activity through chemical homeostasis mechanisms involving inward K+ currents or neurotransmitter clearance at the synaptic cleft ^29,50.^ Finally, astrocytes could alter neuronal activity using their more traditional support mechanisms such as metabolic clearance and neurovascular coupling ^29,51.^ While further studies out of the scope of the present work are needed to identify the exact regulatory mechanisms involved, our data suggests a dynamical link between astroglial-neuronal networks during sensory information processing in the cortex.”** (P. 14, l. 371).

*Reviewer #3:*

*Lines and colleagues explore the role of astrocytic Ca2+ signalling in the processing of sensory stimuli. They start by performing Ca2+ imaging in astrocytes of the somatosensory cortex in anesthetized mice while stimulating the hind paw electrically. They first demonstrate that somatic regions of the astrocytes and their arborization respond reliably and that the responses increase with stimulation duration, frequency and intensity. The simultaneously recorded ECoG displays a similar dependency on the stimulation in the alpha to gamma bands. Interestingly, the astrocyte Ca2+ signals follow the ECoG gamma activity with a delay and terminate before the end of the stimulation and the gamma activity increase. This relationship appears to be altered in IP3R2 knockout animals. Experimental stimulation of astrocyte Gq signalling using DREADDS dampens the gamma activity increase in response to stimulation. These experiments clearly show that astrocytic Ca2+ signalling changes after sensory stimulation are correlated with ECoG activity pattern changes and that manipulating astrocytic Ca2+ signalling affects the gamma response. Overall, this is a very interesting and timely study with elegant experiments and with the important and new result that astrocytic Ca2+ signalling shapes neuronal cortical responses triggered by activation of somatosensory inputs.*

We thank the reviewer for the very positive appraisal of the study and the helpful comments and suggestions that have helped to improve the manuscript.

*Major comments:*

1) *The analysis of imaging data is incompletely described.*

The description of the imaging analysis has now been expanded in Materials and Methods section. We have added a **new Supplementary Figure 8** and the following paragraph that includes a more in-depth description on the selection criteria for astrocyte inclusion in the analysis, how the Matlab algorithm defined astrocyte regions of interest over SR101-stained structure, and how semi-automatic cellular segmentation was performed: **“Images of SR101-stained astrocytes were used to outline individual astrocyte territories. Selected astrocytes had to be in the focal plane with a fully visualized soma and arborization, not cut off at the edge of the image, or overlapped with vasculature. Images of SR101-stained astrocytes were used to outline individual astrocyte territories using a custom MATLAB program (Calsee:** [**https://www.araquelab.com/code/)**](https://www.araquelab.com/code/))**. Calsee identifies astrocyte structure using a strict thresholding of SR101-positive pixels to form a conservative estimate of astrocyte morphology. Astrocyte territories were further segmented into soma and arborization regions of interest (Supplementary Fig. 8). Individual astrocytes were brought to polar coordinates centered at their soma, which was outlined as the radial region with limits defined by 50% of structural fluorescence. Arborization of astrocyte processes was defined as the adjacent remaining radial territory (i.e., outside the soma) whose fluorescence was > 0.25 times the standard deviation over the median of a circle of fluorescence, and represents the ensemble fluorescence of astrocyte processes. Arborization of astrocyte processes were then discretized into a grid of 3 µm x 3 µm square regions, and each of these regions were defined as a microdomain. These structurally-identified regions of interest (i.e., soma, arborization and microdomains) were then used to quantify calcium activity from the simultaneously recorded green channel. Event detection of calcium fluorescence was determined when the amplitude of response was > 3 times the standard deviation of the average baseline amplitude.”** (P. 18, l., 478).

*The authors analyze Ca2+ signals in automatically defined ROIs of 3 x 3 µm2. The illustrated ROIs in Fig. 1b and f do not fill the entire territory of the cell. Why is that? Are only active ROIs shown in these examples?*

As indicated in the new expanded description of the imaging analysis, **“Calsee identifies astrocyte structure using a strict thresholding of SR101-positive pixels to form a conservative estimate of astrocyte morphology.”** (P. 18, l., 482).

*In Fig. 1h, Ca2+ traces from microdomains are shown (pink). The authors need to explain and illustrate with examples, how microdomains, arborization and somatic ROIs were defined. Although the authors show microdomain events (Fig. 1h) these never appear be an analysed in depth. Or are they later pooled with all other transients to calculate the active percentages?*

Following the reviewer´s suggestion, we have included a new **Supplementary Figure 8** to illustrate the procedure to select and identify soma, arborization and microdomains.

Figure 1h aims to illustrate that calcium events occurred at discrete microdomains and could be detected in vivo in response to sensory stimulation. We agree with the reviewer that microdomain event analysis was not further pursued because the rest of the analysis focused on population analysis at the network level, and the analysis centered on arborization, which includes microdomains as guessed by the reviewer. We believe that in depth-analysis of microdomain events is out of the scope of the present work that focused on cellular and network scales.

2) *To fully interpret the ECoG data from IP3R2 knockout mice and DREADD experiments, it is important to know how these manipulations affect the Ca2+ signals under baseline condition and during sensory stimulation. Doing both sets of experiments might not be feasible. It is important however for the IP3R2 knockouts because there is a significant dampening of gamma later during the stimulation*

*(Fig. 4g) and a significant increase of gamma activity during the baseline period (Fig. 5f). Given the variable results in the literature regarding the dependence of (microdomain) Ca2+ signals in astrocytes on IP3R2 it is important to include the microdomain category in the analysis throughout.*

We thank the reviewer for the comment.

Regarding IP3R2^-/-^ mice, we have now included the **new supplementary Figure 3** reporting the analyses of the baseline and sensory-evoked astrocyte Ca^2+^ signals in IP3R2^-/-^ mice. In agreement with previous reports (Takata et al., J Neurosci 2011; Perez-Avarez et al., J Neurosci 2014; Monai et al., Nat Commun 2016), we now report that astrocyte activity in IP3R2^-/-^ mice was strongly diminished in microdomains and negligible at arborizations and somas during both baseline and sensory stimulation (cf. Agarwal et al., Neuron, where relatively infrequent and localized events were only detected in microdomains), which support the validity of IP3R2^-/-^ mice as a loss-of-function approach. The following detailed description has been added to the Results section: “**To test this idea, we analyzed the changes in stimulus-evoked gamma activity in IP3R2^-/-^ mice, in which calcium activity is largely impaired in astrocytes ^23^. We first confirmed in parallel experiments that IP3R2^-/-^ mice displayed strongly diminished astrocyte activity at the microdomain level both in basal and during sensory stimulation (wildtype mice: 1.6 ± 0.6 and 3.1 ± 0.4 events/min in basal and during stimulation, respectively in; p < 0.01; IP3R2^-/-^ mice: 0.8 ± 0.2 and 1.1 ± 0.2 events/min in basal and during stimulation, respectively; p < 0.01; n = 5 imaging planes, 2 animals; Supplementary Fig. 3n). Moreover, astrocyte calcium activity was negligible at the network scale of arborizations and somas (wildtype mice arborizations: 1.0 ± 0.3 and 3.1 ± 0.2 events/min in basal and during stimulation, respectively; p < 0.001; IP3R2^-/-^ mice arborizations: 0.2 ± 0.1 and 0.5 ± 0.2 in basal and during stimulation, respectively, p = 0.15; n = 5 imaging planes, 2 animals; wildtype mice somas: 1.1 ± 0.3 and 2.7 ± 0.2 events/min in basal and during stimulation, respectively; p < 0.05; IP3R2^-/-^ mice somas: 0.4 ± 0.1 and 0.5 ± 0.1 in basal and during stimulation, respectively, p = 0.18; n = 5 imaging planes, 2 animals; Supplementary Fig. 3o-p). These results, which are in agreement with previous reports (cf. ^15,24-26),^ support the validity of IP3R2^-/-^ mice as a loss-of-function approach.**” (P. 7, l., 185).

Regarding the effects of DREADD activation on the Ca^2+^ signal, we have added the **new supplementary Figure 5** showing that after the application of CNO, astrocyte activity is greatly enhanced. We have added the detailed description in the Results section: “**Intraperitoneal injection of the DREADD selective agonist Clozopine N-Oxide (CNO) ^27^ led to an increase in astrocyte calcium (from 12.1 ± 1.5 % to 45.3 ± 8.5 % active astrocytes; p < 0.05; n = 4 animals; Supplementary Fig 5) compared to control conditions with a virus lacking DREADDs (i.e., AAV8-GFAP-mCherry) (from 13.8 ± 6.5 % to 11.6 ± 4.2 % active astrocytes; p = 0.58; n = 4 animals) or saline injections (from 16.6 ± 2.3 % to 22.3 ± 6.6 % active astrocytes; p = 0.74; n = 3 animals).**” (P. 9, l., 225).

*3) The experiments presented in figures 4 and 5 are very interesting and insightful. The baseline gamma activity is higher in IP3R2 knockouts (Fig. 5f) and this seems to partially occlude further increases during stimulation (Fig. 5g). The authors should compare the gamma baseline levels and the relative gamma activity increases during stimulation between control and DREADD experiments and experiments using IP3R2 knockouts.*

We thank the reviewer for the important comment. Following his/her suggestion, we have performed a deeper analysis of the basal and sensory-evoked gamma activity in IP3R2^-/-^ animals and mice with DREADD-expressing astrocytes. First, we have fitted the data presented in Fig. 5 to the equation [2] to obtain the parameters that define the stimulus-dependency of the responses in control (i.e., before CNO or wildtype mice), after CNO and in IP3R2^-/-^ mice (Fig. 5e,g). The results are reported in the **new Table 2**. As pointed out by the reviewer, the results indicate that in IP3R2^-/-^ mice the basal higher gamma activity (denoted by the higher Dmin, Fmin and Imin) occludes further during the sensory stimulation, in other words, the dynamic range of the sensory-evoked gamma activity was reduced in

IP3R2^-/-^ mice. Furthermore, to test whether the astrocyte calcium contributed to the decline of the sensory-evoked gamma activity from the peak to the steady-state, we quantified the steady-state/peak ratio (**new Fig. 4h**). We have added the following paragraphs to describe the results:

- For mice with DREADD-expressing astrocytes: “**Notably, the stimulus-dependence of cortical gamma activity remained, varying as the stimulus duration, frequency, and intensity changed. However, gamma response profiles were significantly reduced across all the stimulating parameters following CNO injections (duration: 2-way ANOVA for stimulus: p < 0.001, for CNO: p < 0.001; frequency: 2-way ANOVA for stimulus: p < 0.001, for CNO: p < 0.001; intensity: 2-way ANOVA for stimulus: p < 0.001, for CNO: p < 0.001; n = 6 animals; Fig. 5e). Indeed, the stimulus-dependent gamma activity curves were shifted to lower values upon CNO application, as indicated by the lower Dmax, Fmax, Imax, Dmin, Fmin and Imin in CNO when compared to control values (Table 2), indicative of a general reduction of the sensory-evoked gamma activity under astrocyte activation. These alterations occur without changes in the astrocyte discrimination or sensitivity to the stimuli, as indicated by the similar values of Dslope, Fslope and Islope, and D50, F50 and I50, respectively (Table 2).**” (P. 9, l. 236)
- For IP3R2^-/-^ mice: "**IP3R2^-/-^ mice showed heightened basal gamma activity compared to wildtype (3.5 ± 1.0 dB in wildtype vs. 9.0 ± 1.8 dB in IP3R2^-/-^ mice; p < 0.05; n = 4 animals; Supplementary Fig. 4), and, like wildtype mice, responded to sustained sensory stimulation with the initial surge of gamma activity that declined to a persistent steady state (Fig. 4f-g). However, while the gamma showed similar peak values in wildtype and IP3R2^-/-^ mice (14.7 ± 0.7 dB and 16.2 ± 1.1 dB respectively; p = 0.30; n = 5 animals each; Fig. 4h), it reached higher steady-state values in IP3R2^-/-^ mice (steady-state: 9.1 ± 0.3 dB in wildtype vs. 12.2 ± 0.7 dB in IP3R2^-/-^; p < 0.01; Fig. 4h). Moreover, the temporal decline of gamma activity during the sensory stimulation was significantly diminished in IP3R2^-/-^ mice, as evidenced by the steady-state/peak ratios (62.2 ± 3.7 % in wildtype vs. 79.5 ± 1.4 % in IP3R2^-/-^ mice; p < 0.01; Fig. 4h). Taken together, these results show that the dynamic range of the gamma activity is reduced in IP3R2^-/-^ mice, and indicate that astrocyte activity regulates the steady-state of the sensory-evoked gamma network activity.**“ (P. 8, l. 200).

*The control data in Fig. 5d-****e (‘before CNO’) and Fig. 5f****-****g (‘wildtype mice’) loo****k identical. If correct, this should be made clear in the legend and labelling.*

Indeed, as guessed by the reviewer, the recordings “before CNO” in wildtype animals served as controls for CNO and IP3R2^-/-^ mice. This is now explicitly stated in the figure legend: “**Note that data of before CNO and wildtype are the same.**” (P. 26, l. 767)

*4) There are a few questions that could be discussed: Why is there a delay of ~ 10 seconds between the onset of the Ca2+ signals and the stimulation? Why does the Ca2+ signal terminate well before the end the stimulation although the effect on gamma is persisting? What cellular mechanisms drive the Ca2+ signals how do they alter gamma activity?*

These are excellent and interesting questions that would require further detailed experimental work, out of the scope of the present study, to be answered. Nevertheless, as indicated by the reviewer, we have added the following paragraph to discuss the issues: “**Multiple signaling pathways may be putatively involved in the bidirectional astrocyte-neuron communication underlying the observed results. On the one hand, astrocytes are known to respond with Ca^2+^ elevations to a wide variety of neurotransmitters released at cortical synapses active during the sensory stimulation, including glutamate, GABA, acetylcholine, norepinephrine or dopamine ^9,11,42.^ On the other hand, multiple astrocyte signaling mechanisms may influence neuronal activity ^9,11,^ either through synapse-specific or volume transmission ^43-45.^ Astrocytes activated during sensory stimulation may release transmitters, such as glutamate ^46,47,^ GABA ^48^, or ATP/adenosine ^22,49,^ that may affect neuronal activity as well as excitatory and inhibitory**

**synapses. Additionally, astrocytes can modulate neuronal activity through chemical homeostasis mechanisms involving inward K^+^ currents or neurotransmitter clearance at the synaptic cleft ^29,50.^ Finally, astrocytes could alter neuronal activity using their more traditional support mechanisms such as metabolic clearance and neurovascular coupling ^29,51.^ While further studies out of the scope of the present work are needed to identify the exact regulatory mechanisms involved, our data suggests a dynamical link between astroglial-neuronal networks during sensory information processing in the cortex.”** (P. 14, l. 371).

Furthermore, **“Present results show interesting aspects of the temporal dynamics of the sensory-evoked astrocyte responses and gamma activity regulation. First, astrocyte Ca^2+^ elevations display a several seconds delay from the onset of the stimulus. The reason for this is unknown, but it is not surprising considering that synaptically-evoked astrocyte Ca^2+^ elevations are largely mediated by the activation of G protein-coupled receptors and IP3R-dependent Ca^2+^ mobilization from internal stores ^9,42,^ which presents relative slower time courses than neuronal activation though ligand- and voltage-gated channels. Second, during the long-lasting stimulation (Fig. 5d), the Ca^2+^ signal subsided while the gamma activity displayed a steady-state. While astrocyte Ca^2+^ did reduce, it remained above baseline until the end of the stimulation similar to gamma levels. Perhaps the downstream signaling processes triggered by the astrocyte Ca^2+^ was persistent or had persistent effects on the neuronal network. Nevertheless, further studies are required to identify the mechanisms that account for these temporal observations.”** (P. 13, l. 359).

*Minor points*

1) *SR101 has been shown to label also oligodendrocytes and to increase neuronal excitability. This should be discussed.*

We agree with the reviewer that SR101 has been shown to label oligodendrocytes as well as astrocytes. However, we believe that the potential labeling of oligodendrocytes would not impact our functional results because they were based on the selective expression of GCaMP6f in GFAP-positive astrocytes. To acknowledge and clarify the issue, we have included the following paragraph in Material and Methods: **“In addition to astrocytes, SR101 has been shown to label oligodendrocytes as well ^60-62.^ However, while we used SR101 labeling to structurally define cells, the cellular Ca2+ activity was assessed using GCaMP6f, which was selectively expressed under the astroglial promoter GFAP, supporting the idea that the recorded calcium activity derives from astrocytes.”** (P. 16, l. 439).

Regarding the potential increase in neuronal excitability by SR101 (Rasmunsen et al., Sci Rep 2016), we used the experiment protocol (50 µM for 20 minutes) that was reported to not induce abnormal activity in sensori-motor cortex (Rasmunsen et al., Sci Rep 2016). We have also clarified the issue in Material and Methods: **“After the dura was removed, sulforhodamine 101 (SR101) was topically applied to the exposed cortex to label astrocyte structure (50 µM for 20 minutes) ^59^, i.e., an experimental protocol that does not affect cortical activity ^59^.”** (P. 16, l. 423).

2) *It would be helpful to mention in the legend of Fig. 2 what D, F and I refer to. Triple asterisks are shown but not explain in the legend. The data is obtained from 11 ‘populations’. How many independent preparations/mice does that correspond to? Have the authors analyzed the delay between the onset of stimulation and the astrocytic Ca2+ signal? I would expect that increasing the stimulation frequency or intensity reduces the delay.*

We thank the reviewer for pointing this out. The figure legend for Fig. 2 has been updated to include the meaning of the fitting parameters D, F an I, and that triple asterisks indicate p<0.001; the number of animals is now reported in the text: **“Fig. 2 Astrocyte population responses are stimulus dependent. a SR101 staining. Scale bar = 50 µm. b Pseudocolor Ca^2+^ images with Ca^2+^ traces of**

**arborizations (*red*) and somas (*blue*) before (basal) and during sensory stimulation with indicated parameters. Scale = ΔF, 10 s. Horizontal black bars indicate sensory stimuli. c Arborization responses vs. stimulus duration (D), frequency (F) and intensity (I). Data were fit to the sigmoid function in equation [1] (see Materials and Methods), where Dmax, Fmax and Imax were the maximum responses for the respective stimulus parameter; Dslope, Fslope and Islope were the respective slopes, which are considered as indicative of the astrocyte discrimination of the sensory stimuli, i.e., how the astrocyte activity response changes as the stimulus changes; and D50, F50 and I50 were the stimulus parameter values at which astrocyte responses were 50% of the maximum values, which was considered as indicative of astrocyte sensitivity of sensory stimuli. d as in *c*, but for somas. Means ± SEM, ‘***’: p < 0.001 ANOVA.”** (P. 25, l. 720).

The question on how astrocytic responses delay would change with the stimulus parameters, is a very interesting idea. As expected by the reviewer, preliminary analysis indicates that increasing the stimulation frequency decreases the response delay. However, we have not pursued further analysis because determining the stimulus-dependency of the onset and decline calcium dynamics would require a detailed and exhaustive analysis that we believe is out of the scope of present work, which focuses on how astrocyte calcium activity regulates neuronal network function.

3) *In the figures 3e and 3f, the values for R2 are very low. This needs to be explained or discussed. Would splitting the data into low and high activity ranges and comparison of the relative delta/gamma activities between those groups give a clearer picture? According to Fig. 2, long/strong/frequent stimuli are associated with a high number of active astrocytes, which is according to Fig. 3e associated with low relative delta activity. What is the explanation that this is not seen in Fig. 3g?*

We thank the reviewer for the comment that prompted us to further consider potential interpretations. We fully agree with the reviewer that R2 values are low (yet, statistically significant). Following the reviewer´s indication we have discussed the issue including the following paragraph in the Discussion: “**Furthermore, our data shows the existence of negative and positive correlation between astrocyte activity and delta and gamma activities, respectively (Fig. 3e and 3f). The statistically significant correlation indicates that these network states are associated with the astrocyte calcium activity, but the relatively low values of the correlation coefficients suggest the involvement of additional factors.**” (P. 12, l. 319).

As suggested by the reviewer, perhaps segmenting data could provide a clearer picture. However, in the absence of a clear rationale to produce certain segmentation, we prefer to report the whole data as is.

We agree with the reviewer that according to Fig. 2 and Fig. 3e, the lack of effects in Fig. 3g is unexpected. However, we believe that the results are not necessarily inconsistent. Indeed, data in Fig. 3e has a much larger statistical power, because the much larger data set derived from all recorded values, than in Fig . 3g, which contains values parsed based on one stimulation parameter and which may therefore fail to show statistical differences.

4) *The demonstration of selective DREADD (mCherry) expression in astrocytes in Fig. 5a is not ideal. Although it clearly shows that neuronal somas are negative for mCherry, it is hard to tell how well the diffuse mCherry signal colocalizes with astrocytic structures. Have the authors quantified the specificity?*

As indicated by the reviewer, we now report the quantification of the immunohistochemistry data, which support the cellular specificity of DREADD expression on GFAP-positive astrocytes: **“The selective DREADD expression by astrocytes in the primary somatosensory cortex was confirmed by immunohistochemistry (Fig. 5a). Immunohistochemical analysis indicated that DREADD expression (monitored by the expression of the fluorescent reporter mCherry) vastly occurred in astrocytes; indeed, while 95.4% of astrocytes were positive for GFAP and mCherry (n = 130**

**cells, 9 slices) only 6.2% of neurons were positive for NeuN and mCherry (n = 259 cells, 4 slices).”** (P. 9, l. 220).

*5) Please specify the frequency bands also in the material and methods section.*

The Materials and Methods section of the revised version now specifies the frequency bands: **“Spectral analysis was performed on the frequency bands of low (0-1 Hz), delta (0-4 Hz), theta (4-7 Hz), alpha (8-12 Hz), beta (13-30 Hz), and gamma (30-50 Hz).”** (P. 19, l. 512).

**Reviewer comments, second version:**

REVIEWERS' COMMENTS:

Reviewer #1 (Remarks to the Author):

The authors have addressed the comments to satisfaction.


Reviewer #3 (Remarks to the Author):

The authors have fully addressed my previous questions and comments.

**Author rebuttal, second version:**

**Reviewer comments, third version:**

**Author rebuttal, third version:**
